# Perspectives on the microorganisms with the potentials of PET-degradation

**DOI:** 10.3389/fmicb.2025.1541913

**Published:** 2025-03-12

**Authors:** Xiao-huan Liu, Jun-li Jin, Hai-tong Sun, Shuo Li, Fei-fei Zhang, Xin-hong Yu, Qi-zhi Cao, Yu-xuan Song, Nan Li, Zhen-hua Lu, Tao Wang, Fei Liu, Jian-min Wang

**Affiliations:** ^1^School of Biological Science, Jining Medical University, Jining, China; ^2^College of Chemical and Biological Engineering, Zhejiang University, Hangzhou, China; ^3^School of Pharmacy, Jining Medical University, Rizhao, China

**Keywords:** poly(ethylene terephthalate), biodegradation, microorganisms, metabolism, upcycling

## Abstract

Polyethylene terephthalate (PET), a widely used synthetic polymer in daily life, has become a major source of post-consumer waste due to its complex molecular structure and resistance to natural degradation, which has posed a significant threat to the global ecological environment and human health. Current PET-processing methods include physical, chemical, and biological approaches, however each have their limitations. Given that numerous microbial strains exhibit a remarkable capacity to degrade plastic materials, microbial degradation of PET has emerged as a highly promising alternative. This approach not only offers the possibility of converting waste into valuable resources but also contributes to the advancement of a circular economy. Therefore in this review, it is mainly focused on the cutting-edge microbial technologies and the key role of specific microbial strains such as *Ideonella sakaiensis* 201-F6, which can efficiently degrade and assimilate PET. Particularly noteworthy are the catalytic enzymes related to the metabolism of PET, which have been emphasized as a sustainable and eco-friendly strategy for plastic recycling within the framework of a circular economy. Furthermore, the study also elucidates the innovative utilization of degraded plastic materials as feedstock for the production of high-value chemicals, highlighting a sustainable path forward in the management of plastic waste.

## 1 Introduction

Nowadays, the world is going through the “Plastic Age,” and the huge surge in the global production of plastics was regarded as the results of their extensive use in the daily life due to fabulous durability and low cost. However, over the past decades, the low biodegradability of plastics has led to a growing concern caused by their detrimental impacts on the natural ecosystem, for instance, the proliferation of plastic waste in the oceans (Ostle et al., 2019; Abel et al., 2023) and the emergence of hazardous microplastics (Lee et al., 2022; Arpia et al., 2021). Although annual single-use plastic generation is still growing exponentially, it was estimated that only ~18% of the plastic waste has been recycled all over the world. Consequently, the plastic waste management has become a critical global issue, which requires innovation to mitigate. Traditional methods including physical cycling (landfilling or mechanical recycling) (Ragaert et al., 2017), thermal treatment (incineration, pyrolysis, gasification, hydrothermal liquefaction), chemical recycling (hydrolysis, glycolysis, aminolysis, methanolysis) (Gao Z. et al., 2022), and biological depolymerization and upcycling (Amalia et al., 2023) have been already proposed and investigated for efficient PET-processing.

**Figure 1:**
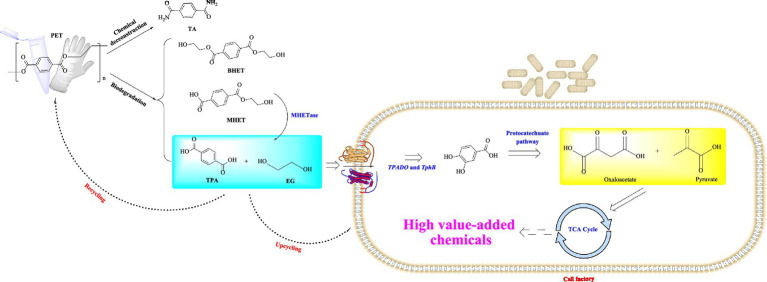
Summary of the established PET-biodegradation pathways. TA, terephthalamide; TPA, terephthalic acid; TPADO, terephthalate 1,2-dioxygenase; TCA, tricarboxylic acid.

PET-biodegrading, facilitated by the hydrolysable ester bonds within its structure, is usually characterized by eco-friendly with low energy consumption, and it also holds significant potential for the upcycling of PET and the recovery of monomers (Singh Jadaun et al., 2022; Qiu et al., 2024). Moreover, the growing significance of the circular economy has further made PET-biodegradation as one of the global research focuses. The objective of PET-biodegradation is biological conversion of recalcitrant wastes to non-toxic lower molecular mass compounds that can enter into the biogeochemical cycle (Klauer et al., 2024). As a primary approach in PET biodegradation, microbial-mediated PET degradation primarily involves four main metabolic pathways: the biodeterioration, bio-fragmentation, assimilation and mineralization (Amobonye et al., 2021). Considering that the PET macromolecular polymers are unable to directly enter the microbial cells, the initial degradation of PET into monomeric products primarily relies on extracellular enzymes secreted by the host microorganisms. Hence, it is considered as the first rate-limiting step. These microorganisms are capable of utilizing different enzymatic systems (Figure 1) (Sui et al., 2023; Liu et al., 2023) to biodegrade PET into smaller water-soluble monomeric products such as terephthalic acid (TPA), monoethylene glycol (MEG), mono (2-hydroxyethyl) terephthalate (MHET), and bis(2-hydroxyethyl) terephthalate (BHET). These monomers could be further utilized by ways of either being metabolized into the tricarboxylic acid cycle (TCA cycle) or being converted into high value-added chemicals (Dissanayake and Jayakody, 2021; Satta et al., 2024). With the rapid development of synthetic biology and metabolic engineering, recently great advance has been made on the bio-cycling of PET waste such as in the fields of the rational engineering of PET-degrading enzymes, reconstruction and optimization of the degradation pathways in microbial chassis (Qi et al., 2021). Extensive evidence suggests that development of optimized microbial chassis and the construction of synthetic microbial consortia, which integrate PET biodegradation through secreted hydrolases with the bioconversion of monomers into high-value chemicals, represents a highly promising strategy for achieving a circular economy in PET waste management (Wang et al., 2025; Diao et al., 2023; Gao R. et al., 2022).

Current studies on PET biodegradation mainly focus on the development of specific enzymes or rational design of special cell-factory capable of PET-biodegradation, and the general aspects of biodegradation mechanisms of plastic-degrading microorganisms without detailed analysis of specific microbials and their unique capabilities. This review offers an in-depth analysis of the current research landscape on the mechanisms of microbial-mediated PET biodegradation, including the enzymes (and the catalytic mechanisms if possible), and the metabolic pathways. It concurrently explores the construction of chassis cells for the efficient biological upcycling of PET, and emphasizes the rational bioengineering of specialized PET-degrading enzymes to boost their performance. Finally, it highlights the challenges in microbial PET-degradation and the future research directions for developing cutting-edge PET recycling technologies. This review is anticipated to serve as a valuable reference for researchers seeking to advance the field of PET biodegradation and facilitate the development of sustainable plastic waste management strategies.

## 2 The wild PET-degrading microorganisms

Despite the physiochemically recalcitrant nature of PET, various microbes capable of plastic biodegradation have been successfully identified in nature such as the *Comamonadaceae* (Knott et al., 2020) and the phylum *Actinobacteria* (Delacuvellerie et al., 2019), microalgae (Barone et al., 2024), bacteria (Yoshida et al., 2016), fungi (Ahmaditabatabaei et al., 2021). Usually, these microorganisms could adhere to the PET surface where they develop consortiums with various microbial species. They are capable of utilizing PET as a major carbon and energy source for its growth such as the well-known *Ideonella sakaiensis* 201-F6 (Yoshida et al., 2016). In this part, several special microorganisms with PET-degrading potential are reviewed, including the catalytic enzymes or the catalytic mechanism if possible.

### 2.1 Prokaryotic cells

#### 2.1.1 Deinococcus maricopensis

PET, one of the most common types of plastic waste, is particularly difficult to degrade due to its crystalline structure, therefore the discovery of thermophilic enzymes capable of breaking down PET has opened new avenues for research. A novel thermophilic polyesterase enzyme, DmPETase, derived from the bacterium *D. maricopensis* was identified recently (Makryniotis et al., 2023). This enzyme is similar to the well-known LCC^ICCG^ (Tournier et al., 2020), which is known for its effectiveness in degrading PET. In this regard, it highlights the importance and potential applications of *D. maricopensis* in the field of PET biodegradation.

**Figure 2:**
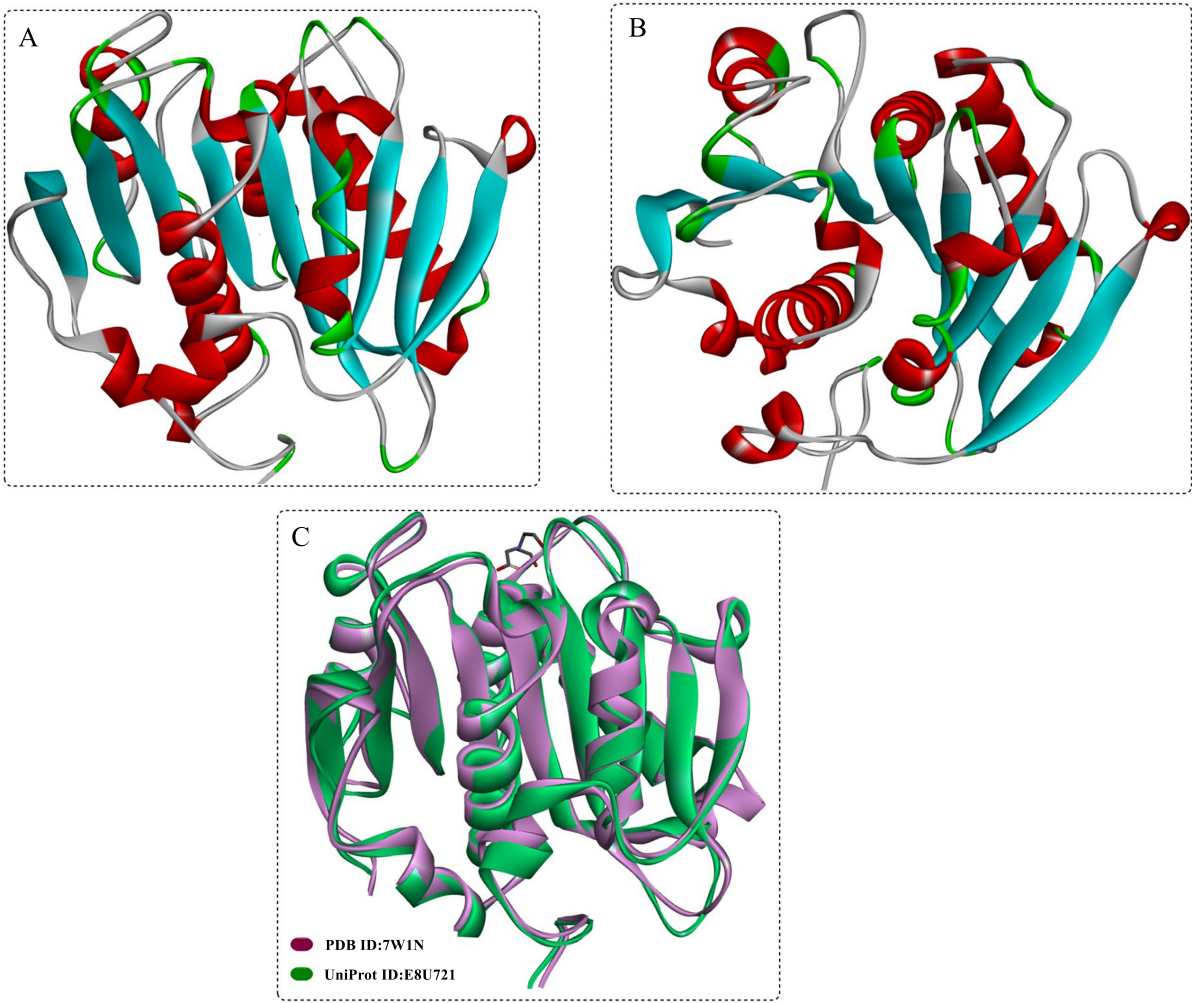
Crystal structures of the DmPETase **(A,B)**, and the structural alignment of DmPETase with the leaf-branch compost cutinase variant LCC^ICCG^ **(C)**.

Structurally (Figures 2A,B), DmPETase is similar to the cutinase-like enzymes especially with a RMSD values of 0.737 Å with LCC^ICCG^ (Figure 2C) and 0.644 Å with *Is*PETase. It also features a catalytic triad (S185-H263-D231) and a single disulfide bond (C296-C312) that classifies it among PET hydrolases, with differences in disulfide bonding patterns distinguishing type I and type II PET hydrolases. The substrate-binding clefts of DmPETase and LCC^ICCG^ show significant similarity, however DmPETase displays a notably wider (10.9 Å) binding site compared to LCC^ICCG^. DmPETase exhibits a distinct electrostatic surface that is mostly neutral with specific negatively charged regions, and compared with the surface mutations in LCC^ICCG^ with the enhanced PET degradation, it suggests that enzyme surface charge could significantly impact PET-degrading capabilities.

DmPETase exhibits thermophilic properties and is capable of degrading synthetic polyesters like PET and polyurethane, as well as semi-crystalline aliphatic polyesters. Similar to LCC^ICCG^, DmPETase could also efficiently degrade amorphous PET, releasing substantial water-soluble products (119 μg/mg_PET_ for DmPETase and 414 μg/mg_PET_ for LCC^ICCG^). Although, the PET-depolymerization performance declines with increasing PET crystallinity, DmPETase and LCC^ICCG^ still manage to degrade semi-crystalline PET, releasing 30.3 μg/mg_PET_ and 35.0 μg/mg_PET_ of hydrolysis products, respectively. Though LCC^ICCG^ is widely acknowledged for its superior efficiency in degrading PET compared with DmPETase, its efficacy tends to wane when dealing with highly crystalline forms of PET. However, when it comes to the degradation of the most crystalline PET, both enzymes exhibit nearly identical effectiveness.

These findings underscore the potential of DmPETase as a promising candidate for enhanced biodegradation of PET waste, particularly in the semi-crystalline state. As research continues to explore the capabilities of DmPETase and other similar enzymes, the prospect of more effective and sustainable solutions to plastic pollution would become increasingly promising.

#### 2.1.2 Streptomyces

Though the increasing accumulation of PET has become a significant environmental concern, the search for biological solutions to degrade PET has led to the identification of various enzymes with potential applications in waste management. Among these, the enzyme Sub1, identified in the genome of *Streptomyces scabies*, has emerged as a promising candidate due to its versatility and efficiency in hydrolyzing a range of natural and synthetic polymers (Jabloune et al., 2020). For example, it could efficiently break down *p*-nitrophenyl esters especially those with short carbon chains. Sub1 also demonstrated hydrolytic activity on the recalcitrant polymers cutin and suberin releasing fatty acids. Sub1 also showed degrading activity on the synthetic polymer PET, as evidenced by the release of TPA. Moreover, the catalytic activity of Sub1 on PET could be significantly enhanced by the addition of Triton, which might be caused by several effects such as the improved surface accessibility, substrate plasticization, interfacial activation, dispersion effect and so on. In addition, Sub1 was shown to be stable at 37°C for at least 20 days, which further highlights its potential for industrial applications. Further research and development could potentially harness capabilities of Sub1 to contribute to sustainable waste management solutions, especially as a robust candidate for industrial-scale applications.

#### 2.1.3 Rhodococcus pyridinivorans

The escalating problem of PET pollution has intensified the search for effective biodegradation methods, and currently it has focused on identifying and characterizing enzymes capable of degrading PET to develop sustainable solutions for efficient PET waste management. A notable discovery in this field is the membrane-anchored esterase from *Rhodococcus pyridinivorans* P23, which has demonstrated efficient PET degradation capabilities (Guo et al., 2023). The average nucleotide identity value reaches to 95.16% between strain P23 and DSM44555. When cultivated with the PET film as sole carbon and energy source for 5 weeks, about 4.28% weight loss (4.03 mg PET) could be achieved with TPA and MHET released as the main products. Genome sequencing and enzymatic characterization showed that the PET-degrading activity result from the PET esterase (OQN32_06240) on the cell membrane of *R. pyridinivorans* P23. Structural analysis indicated that OQN32_06240, harboring a 13–35 amino acid residues transmembrane domain, was most identical to the thermophilic esterase 2 (EST2). OQN32_06240 belongs to the subfamily of the Abhydrolase_3, and the catalytic triad (S175-D277-H313) and GXSXG motif were also determined. At 30°C and under acidic conditions (pH 3.0–4.5), the whole-cell biocatalyst (OD_600_ = 1.0) functions best, which could efficiently degrade the PET substrate (four pieces of PET film, 1.0 cm × 1.0 cm × 0.013 mm, Good Fellow GF89357619) with 1.5 μM compounds (94.0% TPA and MHET) released after 60 h. The *K*_cat_ and *K*_m_ values of this esterase were also determined as 1.63 M^−1^ S^−1^ and 0.102 M^−1^ S^−1^ against BHET and MHET at pH 4.5, 30°C, respectively.

**Figure 3:**
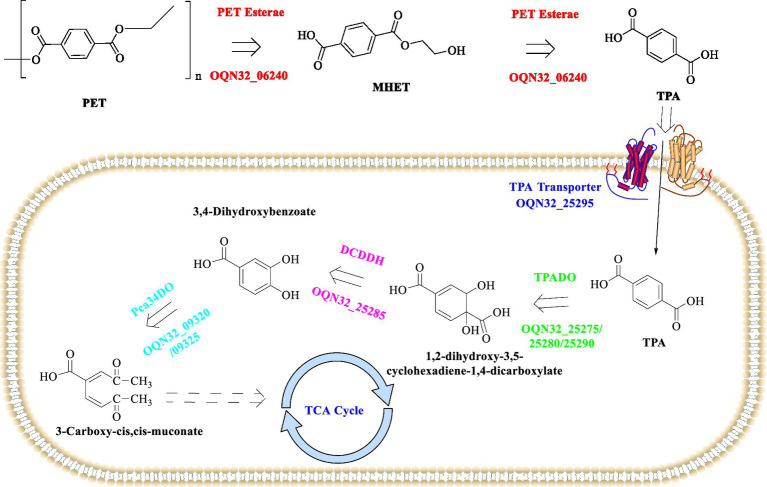
Detailed metabolic pathways associated with PET degradation process in *R. pyridinivorans* P23.

It is found that OQN32_06240 is capable of firstly degrading PET into mainly MHET and tiny BHET, after that the released MHET could be further hydrolyzed into TPA in acidic conditions. The released TPA would then be incorporated into the host cells through the TPA transporter (PcaK) and further metabolized into protocatechuic acid (PCA) catalyzed by TPA 1,2-dioxygenase (TPADO) and 1,2-dihydroxy-3,5-cyclohexadiene-1,4-dicarboxylate dehydrogenase (DCDDH) (Figure 3). However, the aforementioned enzymes along with a regulatory protein named PcaR are encoded by a cluster of genes located on the pA plasmid. The resultant PCA would then enter the tricarboxylic acid cycle, which was catalyzed by the enzymes encoded by the operon (OQN32_22690-OQN32_22715) located on the chromosome. Though the catalytic performance of *R. pyridinivorans* P23 was lower than that of *I. sakaiensis* 201-F6 for the PET-biodegradation (Yoshida et al., 2016), the discovery of the PET-degrading esterase from *R. pyridinivorans* P23 presents a significant step forward in the quest for effective bioremediation strategies against PET pollution. The ability to efficiently degrade PET, coupled with the potential for subsequent integration into metabolic pathways to facilitate complete mineralization and valorization of degradation products, underscores its significant potential as a robust biocatalyst for efficient PET up-cycling. Future research endeavors should prioritize comprehensive characterization of the catalytic mechanisms of OQN32_06240, regulatory operon architecture, and associated metabolic networks, which might facilitate the development of novel biotechnological platforms for sustainable recycling of PET waste and circular economy implementation.

#### 2.1.4 Comamonas testosterone

Recently, the use of alkali-resistant bacteria (*C. testosterone* F6) and surfactants (DDS and Tween) to enhance the biodegradation of PET microplastics was investigated (Li et al., 2023). It demonstrated a significant enhancement of whole-cell biocatalyst activity in an alkaline environment with the addition of Tween 20, resulting in noticeable surface alterations and an 11.04% weight loss of the PET substrate within 5 days. Moreover, it also achieved a remarkable enhancement in product concentration, increasing from 0.084 g/L to 0.152 g/L. The determination of degradation products (mainly TA, MHET and BHET) further confirms that use of surfactants in PET-degradation would not disturb the type of final products. Seen from the infrared spectrum, the characteristic peaks of PET were consistently present across all curves, which signifies that the substrate surface retained its PET macromolecular structure even after biodegradation. Therefore, it is suggested that the degradation mechanism might involve a gradual, layer-by-layer peeling process. The improved PET-degrading activity was attributed to the high hydrophilicity of Tween 20, which, upon adsorption to the PET surface, would facilitate the attraction of extracellular enzymes from the fermentation broth to the substrate, thus enhancing bacterial assimilation and the overall biodegradation process.

#### 2.1.5 Rhodococcus

In recent years, the issue of microplastic pollution, especially that of PET microplastics, has also become a global environmental concern. Attentions have also been delved into an innovative approach by combining alkali-resistant bacteria and surfactants, aiming to unlock new possibilities for more effective degradation of PET microplastics. *Rhodococcus* (Zampolli et al., 2019; Nazari et al., 2022), a genus within *Actinobacteria*, is known for its metabolic versatility and potential to degrade a range of organic compounds, including plastics. Recently, bioinformatic analyses were conducted on 669 *Rhodococcus* genomes for the discovery of potential new plastic-degrading enzymes (Zampolli et al., 2022). Results revealed that certain *Rhodococcus* species, particularly *R. erythropolis*, *R. equi*, *R. opacus*, *R. qingshengii*, *R. fascians*, and *R. rhodochrous*, show promise for plastic-degradation. 2,643, 2,464, 1,786, 1,777, 1,640, and 1,215 genetic determinants were predicted and identified, respectively, which are related to polyester biodegradation across different *Rhodococcus* species. The species predominantly exhibit gene products capable of degrading C–C backbone plastics, and over 50% of unique HIT sequences are related to polyester degradation and the rest mostly are associated with PE degradation. *Rhodococcus* species also show a significant potential for degrading various polyesters, in which the highest number of unique HIT sequences are identified for carboxylesterase, PLA-depolymerase, and PHB-depolymerase enzymes. Among the 17 *Rhodococcus* species with the most unique sequences, *R. hoagii*, *R. equi*, and *R. fascians* displayed high scores, particularly for C–C backbone plastics, while other species showed varying scores related to different plastic-degrading enzymes. This study provides insights into the potential role of different *Rhodococcus* species and genes in the metabolism of various polymers.

**Figure 4:**
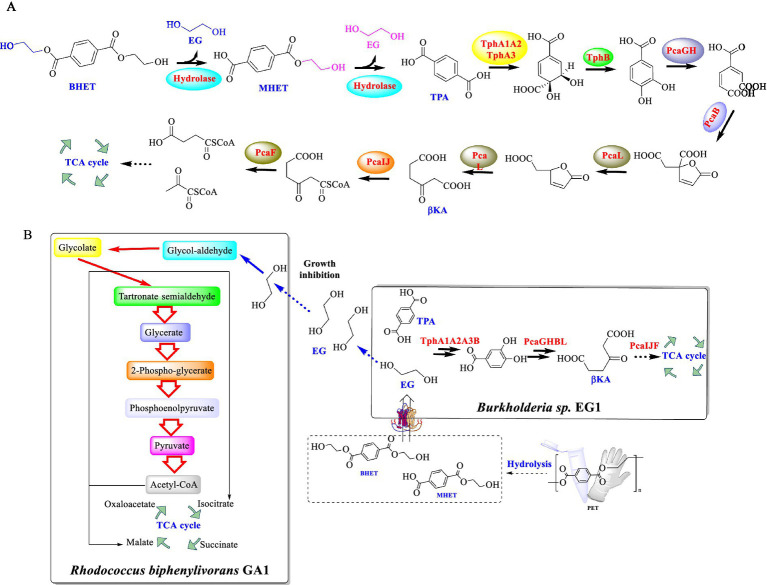
The supposed metabolic pathway of BHET in strain GA1 **(A)**, and the diagram of the metabolism of BETH catalyzed by *R. biphenylivorans* GA1 and *Burkholderia* sp. EG1 co-culture **(B)**.

Recently, the microbial degradation of BHET, a key compound derived from the depolymerization of PET, was also investigated (Jiang et al., 2023). Two strains, *Rhodococcus biphenylivorans* GA1 and *Burkholderia* sp. EG1, were successfully isolated and identified with the ability to utilize BHET as the sole carbon source. Strain GA1 could effectively degrade BHET at 30°C and pH 7.0, with a degradation efficiency of 95% within 18 h. This metabolic pathway was estimated involving the hydrolysis of BHET to MHET and TPA (Figure 4A), followed by further conversion to PCA, β-ketoadipate (βKA), and eventually to acetyl-CoA and succinyl-CoA (Figure 4B). When GA1 and EG1 were co-cultured, EG1 could efficiently metabolize TPA, while GA1 is capable of metabolizing EG. In this way, it could overcome the inhibitory effect caused by the accumulated EG. Moreover, this co-culture system achieved the complete degradation of 10 mM BHET within 72 h. During this process, the growth for both strains was enhanced, which indicates a mutually beneficial symbiotic relationship. Especially, a novel esterase gene, *betH*, was identified from strain GA1, which encodes an esterase displaying BHET-hydrolyzing activity. Under the optimal conditions (at 30°C and pH 7.0), the BetH significant catalytic efficiency toward BHET hydrolysis, demonstrating a turnover rate (*k_cat_*) of 16.7 ± 0.56 s^−1^.

It clearly demonstrated the synergistic effect of using alkali-resistant bacteria (*C. testosterone* F6) in conjunction with surfactants (DDS and Tween) on enhancing PET microplastic biodegradation efficiency. Future attentions might be focused on three critical aspects: (1) the optimization of the bacterial-surfactant combination for maximum degradation efficiency; (2) comprehensive assessment of the long-term ecological impact of the biodegradation process, and (3) development of scalable applications for industrial waste treatment. These investigations could potentially provide a sustainable and environmentally friendly approach to addressing the global challenge of PET microplastic pollution.

In another study, the biodegradation potential of *Rhodococcus erythropolis* MTCC 3951 on TPA was investigated (Maurya et al., 2024). In mineral salt media with TPA as the sole carbon and energy source, it could degrade 5 mM and 120 mM TPA within 10 h and 84 h under the optimal conditions, respectively. Furthermore, enzymatic characterization combined with metabolic profiling provided conclusive evidence for the ortho-cleavage pathway of TPA degradation. This was confirmed through by: (1) quantitative analysis of key enzymatic activities of protocatechuate 3,4-dioxygenase (P34O) and protocatechuate 4,5-dioxygenase (P45O), and (2) comprehensive GC–MS identification of intermediate metabolites along the degradation pathway. Interestingly, metabolic profiling revealed the concurrent biosynthesis of polyhydroxyalkanoates (PHAs) during this process, and this suggested a potential metabolic link between TPA degradation and PHA accumulation in the bacterial strain. Moreover, this bacterium could also effectively degrade TPA in real industrial wastewater, which further displays the potentials for environmental pollution control and bioplastic production.

#### 2.1.6 Pseudomonas

Though biodegradation using microorganisms offers a promising alternative to traditional plastic waste management, finding effective microbial strains and enzymes for degrading resistant plastics remains challenging. Recently, several researches focus on isolating novel microbial consortia and identifying new PET-hydrolases, aimed to develop more efficient and sustainable strategies for plastic waste management. Recently, two microbial consortia, designated ConsPlastic-A and ConsPlastic-B, were isolated from soil at a plastic-contaminated landfill capable of utilizing polyethylene or PET as a carbon source (Roman et al., 2024). Among these, a novel strain, *Pseudomonas putida* BR4, was identified for its ability to degrade PET and produce a PHB-HV copolymer with enhanced properties. The average nucleotide identity of strain BR4 was 91% compared to *P. putida* PICP2 (GenBank: GCA_014269225.2) and 90% compared to *P. anuradhapurensis* (GenBank: GCA_000412675.1). Notably, this strain also displays significant potential for bio-consolidated plastic depolymerization and upcycling.

Recently, two novel PET-hydrolases have been identified: *Pp*PETase, originating from *P. paralcaligenes* MRCP1333 found in human feces, and *Sc*PETase from *Streptomyces calvus* DSM 41452 (Han et al., 2024). They are capable of depolymerizing diverse PET materials, including semicrystalline PET powders (Cry-PET, Goodfellow, US 277-377-58; density: 1.3–1.4 g cm^−3^; nominal granule size: 3–5 mm) and low-crystallinity PET films (gf-PET, prepared in a circular form [6 mm in diameter, ~5 mg]). The PET-degrading activity of *Pp*PETase and *Sc*PETase against Cry-PET was 1.53 and 3.62 times greater than that of LCC at 30°C, respectively. Moreover, the degradation activity of *Pp*PETase toward Cry-PET at 50°C could be increased by 44.28% than that at 30°C. Given these, structure-based rational design was conducted: (1) Engineering of the substrate-binding region of *Pp*PETase (Y250N mutation) improved the degradation of long-chain PET by 2.1-fold, which might be caused by the enhanced mobility of the extended loop and facilitating the accommodation of long-chain PET structures. (2) Combinatorial mutations around the substrate binding pocket led to the identification of the most effective variant *Pp*PETase^Y239R/F244G^, which displays a 4.6-fold improvement in BHET degradation activity. The addition of the Y250G mutation to this variant resulted in a 3.1-fold increase in Cry-PET degradation. (3) Further engineering result in the *Sc*PETase^A212C/T249C/N195H/N243K^ variant, which showed a 1.6-fold increase in BHET degradation and a 1.9-fold enhancement in Cry-PET degradation.

Then co-expression of *Sc*PETase and *Is*MHETase in *E. coli* was achieved, and the obtained engineered cells were used to break down PET. After 30 days, the whole-cell catalysts were capable of depolymerizing Cry-PET by over 40% and gf-PET by over 6%, which led to the production of 37.7% TPA from Cry-PET and 25.6% TPA from gf-PET. This underscores the effectiveness of the co-expression system in breaking down PET into smaller molecules. Given these, future research could be directed toward exploring the large-scale application and the environmental impact of this whole-cell system in industrial waste treatment.

#### 2.1.7 *Bacillus* spp.

Understanding the roles of wild microorganisms in plastic biodegradation and developing new screening strategies to identify the potential strains are crucial steps toward solving the plastic waste problem. The wild *Bacillus* species have been discovered to be instrumental in the biodegradation of plastics, primarily due to the secreted surface-active biosurfactants. These compounds significantly enhance the accessibility of plastics and facilitate the colonization by microorganisms. In a recent study, a pioneering screening strategy was developed using polycaprolactone (PCL) and PET nanoparticles as substrates. This innovative approach led to the discovery of a new strain of *Bacillus safensis*, designated YX850, which exhibits PET-degrading activity (Zeng et al., 2023). Microorganisms are initially screened on LB agar plates supplemented with PCL to identify strains exhibiting hydrolytic activity, as indicated by the formation of transparent halos. Subsequently, PET degradation assays are conducted, followed by comprehensive analysis of PET nanoparticle degradation products to confirm the biodegradation capability. Results indicated that *B. safensis* YX8 is efficient in breaking down PET nanoparticles into BHET, MHET, and TPA. However, it exhibited minimal degradation capacity against PET film, without detectable degradation products (BHET, MHET, and TPA). Nonetheless, the water contact angle (WCA) of the PET surface increased from 69.77° to 79.30°, which suggests an enhanced hydrophobicity. Moreover, results demonstrated that the extracellular enzymes secreted by strain YX8 were capable of forming distinct hydrolysis zones on PCL plates within 12 h of incubation. Quantitative analysis revealed that the extracellular esterase activity reached its peak level at approximately 72 h of cultivation. When used for degradation of PET nanoparticles, TPA (2.9 nM), MHET (0.1 μM), and BHET (22.3 nM) could be detected by HPLC. But no degradation products were produced for the boiled enzymes, which further highlighted the degradation capability of strain YX8 against the PET nanoparticles.

In light of these findings, research efforts should be primarily directed toward optimizing and systematically exploring the catalytic capabilities of strain YX8 to enhance its degradation efficiency across various PET substrates, with particular emphasis on PET film applications. Additionally, investigating the large-scale application of this strain in industrial waste treatment and exploring the genetic mechanisms underlying its degradation ability would also be beneficial.

#### 2.1.8 Penicillium

While microbial biodegradation has emerged as a promising and environmentally sustainable strategy for PET waste management, the discovery and characterization of novel microbial strains with enhanced PET-degrading capabilities continues to represent a critical and rapidly evolving research frontier in environmental biotechnology. *Penicillium* species, which are well-recognized for lipase production, offer an interesting avenue for investigating microbial polyester degradation mechanisms. In a recent study, it focuses on exploring the potential of *P. restrictum* and *P. simplicissimum* in the biodegradation and depolymerization of post-consumer PET waste (Moyses et al., 2021). Results revealed that *P. simplicissimum* achieved maximum lipase production of 606.4 U/L when induced with BHET, representing an approximate two-fold increase compared to the non-induced control condition. After 28 days, *P. simplicissimum* caused a 3.09% mass loss on PC-PET (post-consumer PET, derived from waste bottles of brand Crystal© in the form of square fragments of 5.0 mm sides and 0.1 mm thickness) fragments, while *P. restrictum* resulted in a negligible 0.08% mass loss. Enzymatic depolymerization analysis revealed that *P. simplicissimum* culture filtrates generated limited quantities of BHET, MHET, and TPA, and it suggested that intact cellular systems and organism-level metabolic processes might play a crucial role in enhancing PET biodegradation efficiency. In addition, it was found that MHET was the predominant hydrolyzing product, and this suggested a potential feedback inhibition mechanism caused by MHET accumulation on the enzymatic degradation process. It was believed *P. simplicissimum* is a promising bio-degrader of PC-PET, which is capable of achieving significant mass loss and monomer recovery. Moreover, it was also suggested that further research is needed to understand the mechanisms at the organism level that contribute to PET depolymerization and to optimize the biodegradation process for PET waste management in a circular economy context. Additionally, optimizing the biodegradation conditions, such as temperature, pH, and nutrient availability, can enhance the efficiency of PET waste degradation. These efforts will be crucial for the effective management of PET waste in the context of a circular economy.

#### 2.1.9 *Ideonella sakaiensis* 201-f6

Among the microorganisms capable of bioremediation and bio-recycling of PET, *I. sakaiensis* 201-f6, with its unique ability to degrade and assimilate PET, has drawn significant attention. Understanding the enzymatic mechanisms and metabolic pathways involved in its PET-degrading process is crucial for developing enhanced biodegradation strategies, and engineering more efficient microbial systems. Investigations have comprehensively characterized the enzymatic systems and metabolic networks in *I. sakaiensis* that facilitate the conversion of PET monomers into assimilable intermediates (Yoshida et al., 2016; Hachisuka et al., 2021). Two hydrolytic enzymes were identified, PET hydrolase (*Is*PETase) and MHET hydrolase (MHETase), which are capable of synergistically converting PET into its monomeric building blocks.

However, the other enzymes involved in the conversion of PET into its monomers remain largely unexplored. It was found that in the presence of EG and PET, the *ISF6_0529* gene was significantly upregulated (Yoshida et al., 2016), a phenomenon consistent with the metabolic pathways of EG in other typical organisms, which involve homologs of the ISF6_0529 protein (Hachisuka et al., 2022). Findings also disclosed that the ISF6_0529 protein (*Is*PedI), ISF6_2768 protein (*Is*PedE), and ISF6_2763 (*Is*PedH) show a high degree of sequence identity with *Pp*PedI (68.2%), a homolog of *Pp*PedE (70.4%), and a homolog of *Pp*PedH (64.4%), respectively. This suggests that the EG metabolic pathway in *I. sakaiensis* is analogous to that observed in *P. putida*. It was found that *Is*PedE and *Is*PedH also exhibit EG dehydrogenase activity and are dependent on Ca^2+^ and t Pr^3+^, respectively. The specific activity toward EG was determined to be 1.2 ± 0.0 μmol min^−1^ mg^−1^ for *Is*PedE. *Is*XoxF, another dehydrogenase gene product, was found to be upregulated when the bacteria grow on EG. It also shows slight EG dehydrogenase activity (0.065 ± 0.007 μmol min^−1^ mg^−1^) and is regulated by Pr^3+^. These are consistent with the findings that *Pp*PedE is a Ca^2+^-dependent alcohol/aldehyde dehydrogenase, and *Pp*PedH is dependent on lanthanides as a cofactor. These findings underscore the distinctive alcohol oxidation pathway in *I. sakaiensis*, which efficiently utilizes EG and specifically depends on metal ions for their catalytic activities.

*Is*PedI, as an aldehyde dehydrogenase, displayed highest catalytic activity against glycolaldehyde (1.8 ± 0.1 μmol min^−1^ mg^−1^), which is considered to be a critical enzyme in the metabolic pathway of EG. Moreover, it was found that the expression of EG dehydrogenases in *I. sakaiensis* is also dramatically influenced by rare earth elements (REEs), especially Pr^3+^ was shown to display the most significant regulatory effect on the expression of *Is*PedE, *Is*PedH, and *Is*XoxF, which is similar to the regulatory patterns observed in *P. putida* KT2440.

Structural analysis disclosed that *Is*XoxF possesses a more expansive catalytic pocket than *Is*PedE and *Is*PedH, which might endow it with the capacity to accommodate larger substrates. This is corroborated by the observation that mutations in this region of *Pp*PedH are capable of modulating substrate specificity and catalytic efficiency. Ultimately, the growth of genetically engineered strains with targeted gene disruptions underscored the pivotal role of *Is*PedE in EG-mediated growth, and highlighted the enhanced significance of *Is*PedH and *Is*XoxF in the presence of Pr^3+^. The Pr^3+^-mediated restoration of growth in a strain deficient in *Is*PedE implies that Pr^3+^-induced enzymes, such as *Is*PedH and *Is*XoxF, can compensate for the absence of *Is*PedE. Nonetheless, the diminished growth rate and cell yield in the absence of *Is*PedE suggest its superior roles in EG metabolism. The growth characteristics of the double disruption strain (P1-*ΔIspedEΔIspedH*) suggest that additional Pr^3+^-induced EG dehydrogenases, including *Is*XoxF, may also participate in this metabolic process. Pr^3+^ negatively regulated *Is*PedE and positively regulated *Is*PedH and *Is*XoxF, which indicates a switch in enzyme function in the presence of REEs. These findings proposed a model where *I. sakaiensis* alternates between *Is*PedE and the Pr^3+^-induced *Is*PedH and *Is*XoxF for EG metabolism, highlighting the adaptability of *I. sakaiensis* in different environmental conditions.

These studies have provided in-depth insights into the metabolic pathways and enzyme functions in *I. sakaiensis* 201-f6 during the conversion of PET monomers. It not only contributes to the fundamental knowledge of PET-degrading microorganisms but also has practical implications for bioremediation and bio-recycling of PET waste. Further genetic engineering techniques could be employed to modify the bacterium and develop efficient cell-factory to better adapt to different environmental scenarios. This would pave the way for more sustainable solutions to the global PET pollution problem.

#### 2.1.10 *Delftia* sp. WL-3 species

To address the issue of the accumulation of plastic waste, the identification of novel bacterial strains capable of degrading PET and its monomers offers a promising approach to mitigate plastic pollution. In a study, the biodegradation capabilities of a newly identified bacterial strain, *Delftia* sp. WL-3 was investigated, which shows potential in breaking down both diethyl terephthalate (DET) and PET (Liu et al., 2018). *Delftia* sp. WL-3 was isolated from activated sludge and identified using 16S rRNA gene sequencing. *Delftia* sp. WL-3 grew well on both DET and PET, and especially it could degrade 94% of 5 g·L^−1^ DET within 7 days as the sole carbon source. Significant surface damage on the PET film were identified after inoculation with strain WL-3, and further confirmed by SEM analysis, which demonstrated potentials for PET bioremediation. Moreover, Strain WL-3 showed stable DET degradation over a wide range of pH (6.0–9.0) and temperatures (20–42°C), with optima at pH 7.0 and 30°C, which was determined that this process follows first-order kinetics with a half-life of 78 h. Analysis of the intermediates suggested that DET could be directly degraded to TPA, and PET was firstly hydrolyzed to MHET, which was further oxidized to TPA and then to protocatechuic acid (PCA). These findings demonstrate that *Delftia* sp. WL-3 is a robust bacterial strain capable of degrading both DET and PET under various environmental conditions, which clearly highlights its promise for bioremediation applications. Especially, the identification of degradation intermediates provides valuable insights into the biodegradation pathways of PET, which can inform future research and the development of strategies for PET management.

#### 2.1.11 *Cutinase*-producers (*Humicola insolens*, *Thielavia terrestris*, *Pseudomonas mendocina*, *Penicillum citrinum, T. cellulosilytica, T. fusca*, and *T. alba*)

Cutinases, a unique group of esterases, have shown great potentials in the hydrolysis of PET. Understanding the catalytic mechanisms and enhancing the performance of cutinases from different microbial sources are crucial steps toward the development of efficient PET-recycling technologies. This part focuses on the exploration of various cutinase-producing microorganisms and their potentials in PET degradation, aiming to provide new insights and strategies for the bioremediation of PET-polluted environments.

Cutinases sourced from a variety of *Thermobifida* species, namely *T. cellulosilytica*, *T. fusca*, and *T. alba*, exhibited remarkable hydrolytic activity toward PET. Nonetheless, their catalytic activities are primarily mediated by the enzyme hydrophobicity and the electrostatic properties encircling the substrate-binding cleft. To improve the catalytic performance, cutinases from *T. fusca* (TfCut2) (Mrigwani et al., 2023) and Leaf and branch compost cutinase (LCC) (Tournier et al., 2020) have been subjected to mutation to enhance their surface hydrophobicity resulting in the mutants exhibiting improved PET hydrolytic performance.

In addition, cutinases originating from other microbial sources have also been identified to facilitate the hydrolysis of PET (Sui et al., 2023), including enzymes from *Humicola insolens*, *Thielavia terrestris*, *Pseudomonas mendocina*, and *Penicillium citrinum*. This highlights the diversity of microorganisms with PET-degrading activity. Notably, the cutinase from *Humicola insolens* (HiC) has displayed high hydrolytic activity due to its stability at 70°C, a temperature close to the glass transition temperature (*T_g_*) of PET. The increased temperature might enhance the accessibility of HiC to PET ester groups, as the chain mobility in the amorphous phase is higher at this temperature. For instance, the commercial HiC enzyme (Novozym® 51032) demonstrated superior performance in the depolymerization of PET into TPA.

To reduce dependency on the energy-intensive process of PET melt-amorphization, Karine et al. suggested that HiC could effectively depolymerize crystalline PET powder (with 36% crystallinity) in moist-solid reaction mixtures without the need for any prior treatment. The PET substrate could be efficiently degraded into TPA at 55°C after 7 d (20 ± 1% yield), with a 20-fold selectivity over the MHET and BHET. This exceeds the results achieved under the conventional aqueous conditions with a 10% yield and only 2.8-fold selectivity for TPA over MHET. Similar findings were also reported for the hydrolysis of postconsumer PET bottles (30–35% crystalline) with the obtained yields of TPA 16 ± 2% (transparent), 13.0 ± 0.4% (green), and 14.4 ± 0.7% (blue), respectively. Moreover, the catalytic performance could be further improved by reactive aging (5 min ball milling at 30 Hz and 24 h aging at 55°C), which would lead to a 21 ± 2% yield of TPA in only 3 d. To tackle the issue caused by the decreasing activities of surface-bound HiC (~96% of initial activities lost), the enzyme was supplemented in batches. This strategy achieved an overall 49 ± 2% yield of TPA from 900 mg of PET utilizing only a total of 3 wt% of enzyme after 7 rounds. This strategy provides a new perspective for PET-recycling, advocating for a more sustainable and environmentally friendly approach under milder conditions. However, it is necessary to determine the scope of application of this method, and whether it is equally applicable to the mesophilic enzymes (e.g., *Is*PETase) and thermophilic enzymes (e.g., LCC). Especially, the full potential of these enzymes should be explored, including their applicability to different types of PET waste and the scalability of the process, which would ultimately contribute to a circular economy and reducing plastic pollution.

#### 2.1.12 Bacteroidetes phylum

The Bacteroidetes phylum also exhibits potential in PET degradation due to its diverse enzymatic capabilities, particularly in breaking down complex polymers. Certain species within this phylum are known to produce esterases and cutinases, which can hydrolyze ester bonds in PET, facilitating its decomposition into smaller, environmentally friendly products. Recently, a PETase-specific Hidden Markov Model (HMM) search algorithm has been employed to explore novel PET-active enzymes within the Bacteroidetes phylum, which holds the potential to uncover new enzymatic candidates (Zhang et al., 2021). Two esterases, PET27 from *Aequorivita* sp. and PET30 from *Kaistella jeonii*, were identified capable of depolymerizing PCL (amorphous PET film) and the polyester polyurethane Impranil®DLN. Results showed that PET27 could effectively catalyze the release of an average of 174.4 nmol of TPA from a PET foil platelet, which represented a 38-fold increase compared to the activity of PET30 under identical conditions. However, the results indicated that the enzymatic activity of *Is*PETase is 4.7 times that of PET27, which led to the speculation that PET may not serve as the optimal substrate for either enzyme. Particularly, at 4°C, over 30 days in a 200 μL reaction volume, PET30 (1 mg/mL) was able to catalyze the production of ~6.1 μM TPA. In contrast, PET27 failed to release any measurable TPA under these conditions. However, *Is*PETase, under the same conditions, enabled the release of TPA in an almost equivalent quantity, with an average of 5.9 μM.

**Figure 5:**
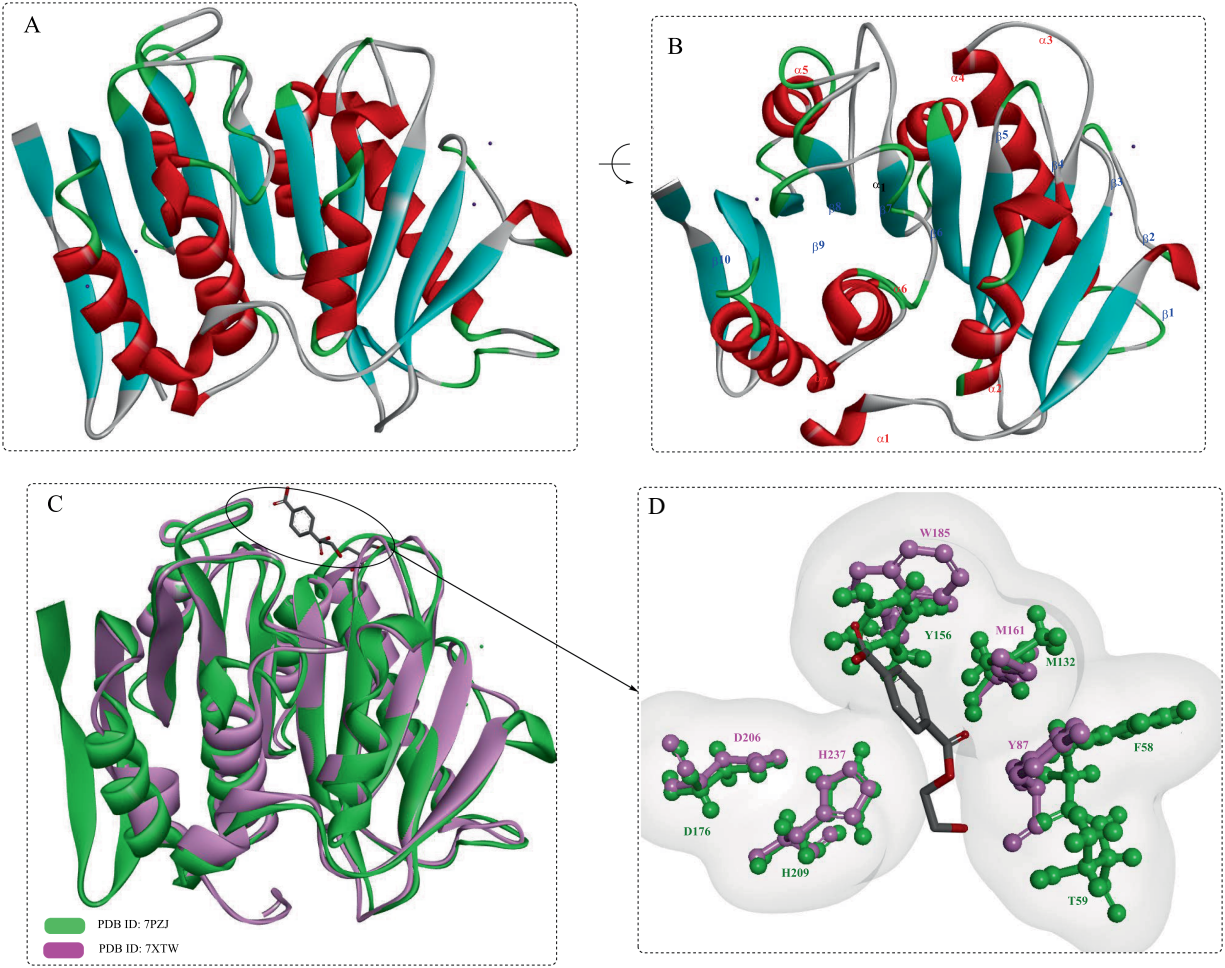
Detailed structural analysis of PET30. **(A,B)** Crystal structure of PET30; **(C)** structural alignment of PET30 and IsPETase; **(D)** the binding modes of MHET in the active sites of PET30 and IsPETase.

Structural analysis of PET30 (Figure 5) demonstrated that, similar to PETases and cutinases, it features a characteristic α/β-fold structure (Figure 5B). It is highlighted by a centrally twisted β-sheet composed of 9 β-strands, flanked by 7 α-helices on both sides. The most notable distinction between PET30 and *Is*PETase is the presence of an extra β-sheet (β10) in PET30 (Figure 5C), which might play a crucial role in connecting with the secretion PorC-like motif. Additionally, the catalytic pocket of PET30 shows a significantly less hydrophobic environment, which potentially affected its substrate specificity and catalytic efficiency as revealed by the biochemical experiments.

Amino acid sequence analysis also unveiled that PET30 possesses a unique Phe-Met-Tyr substrate binding motif (Figure 5D), which is distinct from the binding residues present in *Is*PETase and LCC. In contrast, PET27 shows a Phe-Met-Trp motif identical to that of Cut190. In addition, PET27 and PET30 are likely secreted enzymes, as they possess a C-terminal secretion signal that facilitates transport into the periplasm. In addition, they are also featured by a PorC-like secretion motif, which is associated with the type IX secretion system.

Homologs of PET27 and PET30 were identified in global metagenomes, with a notable prevalence in aquatic ecosystems (predominantly occurring in marine environments), which implies their involvement in the degradation of PET in diverse settings. A substantial number of potential homologs were detected within the Flavobacteria-Cytophaga-Bacteroidetes group, and this further underscores the crucial role of marine microorganisms in the breakdown of plastic materials (Table 1).

**Table 1:** The enzymes utilized for PET degradation.

Type	Organism	Enzyme(s)	Optimal conditions	PET degradation efficiency	Ref.
Prokaryotic cells	*Deinococcus maricopensis*	DmPETase	Thermophilic conditions	amorphous PET: 119 μg/mgPET of hydrolysis products; Semi-crystalline PET: 30.3 μg/mgPET of hydrolysis products	Makryniotis et al. (2023)
	*Streptomyces scabies*	Sub1	Stable at 37°C, enhanced by Triton	Releases TPA from PET	Jabloune et al. (2020)
	*Rhodococcus pyridinivorans* P23	PET esterase (OQN32_06240)	30°C, acidic conditions (pH 3.0–4.5)	4.28% weight loss in 5 weeks	Guo et al. (2023)
	*Comamonas testosterone* F6	/	Alkaline environment with Tween 20	11.04% weight loss of PET substrate within 5 days	Li et al. (2023)
	*Rhodococcus* spp. (e.g., *R. erythropolis, R. equi*, etc.)	/	/	*R. erythropolis* MTCC 3951: TPA degradation; *R. biphenylivorans* GA1: BHET degradation	Zampolli et al. (2022), Jiang et al. (2023), Maurya et al. (2024)
	*Pseudomonas putida* BR4	/	/	Degrades PET and produces a PHB-HV copolymer	Roman et al. (2024)
	*Pseudomonas paralcaligenes* MRCP1333	PpPETase	50°C	Against Cry-PET at 30°C: 1.53 times greater activity than LCC; at 50°C, 44.28% increase in degradation activity compared to 30°C	Han et al. (2024)
	*Streptomyces calvus* DSM 41452	ScPETase	/	Against Cry-PET at 30°C: 3.62 times greater activity than LCC	Han et al. (2024)
	*Bacillus safensis* YX850	/	/	Degrades PET nanoparticles; no detectable activity for PET film	Zeng et al. (2023)
	*Penicillium restrictum*	/	/	0.08% mass loss on PC-PET fragments after 28 days	Moyses et al. (2021)
	*Penicillium simplicissimum*	/	BHET induction for lipase production	3.09% mass loss on PC-PET fragments after 28 days	Moyses et al. (2021)
	*Ideonella sakaiensis* 201-f6	PET hydrolase (*Is*PETase), mono(2-hydroxyethyl) terephthalate hydrolase (MHETase), *Is*PedE, *Is*PedH, *Is*XoxF	/	111	Yoshida et al. (2016), Hachisuka et al. (2021), Yoshida et al. (2016), Hachisuka et al. (2022)
	*Delftia* sp. WL-3	/	pH 7.0, 30°C	Degrades 94% of 5 g·L^−1^ DET within 7 days with significant surface damage on PET film	Liu et al. (2018)
	*Aequorivita* sp.	PET27	/	Catalyzes release of an average of 174.4 nmol of TPA from a PET foil platelet	Zhang et al. (2021)
	*Kaistella jeonii*	PET30	4°C	Catalyzes production of ~6.1 μM TPA in 30 days	Zhang et al. (2021)
Eukaryotic cells	*Yarrowia lipolytica* IMUFRJ 50682	/	With 5 wt% watermelon peels for PET degradation; 450 rpm agitation for GA production	Yields up to 42.02 mmol·L^−1^ TPA with 5 wt% watermelon peels; highest TPA concentration of 21.2 μmol·L^−1^ in 7 days with 20 wt% PET and apple peels + commercial cork	de Souza et al. (2020)
	*Microsphaeropsis arundinis* CBMAI 2109, CBMAI 2110	/	/	CBMAI 2109: 0.5% reduction in PET mass after 14 days; CBMAI 2110: 0.16% reduction in PET mass after 14 days; degrade PET nanoparticles into TPA with rates of 2.0 ± 0.4% and 2.7 ± 0.9%	Malafatti-Picca et al. (2019)
	*Candida antarctica*	Lipase B (CALB)	Acidic conditions (pH 5) for complete hydrolysis of BHET to TPA and EG; neutral-to-alkaline conditions (pH 7 and 9) for selective hydrolysis to MHET and EG	Capable of hydrolyzing triacylglycerides and depolymerization of polyesters (e.g., polylactic acid, small PET oligomers).	Świderek et al. (2023)
	*Aspergillus oryzae*	Cutinase (AoC)	/	Nearly complete degradation (87%) of PCL films within 6 h; higher thermostability (*T*_m_ = 59°C)	Liu et al. (2009)
Eukaryotic Cells (Human-related)	human saliva	MG8 (from human saliva metagenome)	55°C (optimal for TPA production)	Converts PET powder into detectable levels of MHET (26-fold increase) and TPA (3-fold increase) compared to IsPETase at 37°C; at 55°C, generates approximately 83 times more TPA than *Is*PETase	Eiamthong et al. (2022)
Plant-associated cells (Rhizobacteria)	*Priestia aryabhattai* VT 3.12, *Bacillus pseudomycoides* VT 3.15, *Bacillus pumilus* VT 3.16	/	/	VT3.12: 40% weight loss of PET sheet over 28 days, 69% weight loss of 300 μm PET powder over 18 days; VT3.15: 36% weight loss of PET sheet over 28 days, 66% weight loss of 300 μm PET powder over 18 days; VT3.16: 32% weight loss of PET sheet over 28 days, 64% weight loss of 300 μm PET powder over 18 days	Dhaka et al. (2023)
Microalgae	*Phormidium lucidum, Oscillatoria subbrevis*	Ligninolytic and exopolysaccharide enzymes	/	Biodegrade LDPE	Sarmah and Rout, 2018
	*Uronema africanum Borge*	/	/	Initiates biodegradation of LDPE within 30 days	Sanniyasi et al. (2021)
	*Spirulina* sp.	/	Saline systems	Degrading PET in the state of water with a salinity 7 ppt.	Hadiyanto et al. (2022)

Moreover, metagenomic analyses confirmed that *K. jeonii* is predominantly present in aquatic habitats and has the ability to colonize PET surfaces after incubation (Zhang et al., 2021). Microscopic analyses further revealed that *K. jeonii* cells can adhere to and colonize PET surfaces, a process that is associated with the functionality of SusD binding modules. This research not only expands the comprehension of PET-degrading enzymes but also pinpoints potential candidates for biotechnological applications, especially in low-temperature environments. Additionally, further studies on the engineering of these enzymes for improved catalytic performance and the analysis and rational regulation of these enzymes in the metabolic pathways are essential for practical application of these potential enzymes for sustainable PET waste management strategies.

### 2.2 Eukaryotic cells

#### 2.2.1 Fungi

Fungal strains, resilient in the face of extreme pH and humidity levels, show greater potentials to assimilate the polymer. This could be largely attributed to the multienzyme system, which could produce various enzymes [the cutinases (Sahu et al., 2023), esterases (Austin et al., 2018), lipases (Hwang et al., 2022) and so on (Temporiti et al., 2022)] facilitating the degradation of different organic matters. For example, recently four PET degrading strains were identified from 100 fungal strains, which are capable of catalytic conversion of PET to TPA (Malafatti-Picca et al., 2023), including *Curvularia trifolii* CBMAI 2111, *Trichoderma* sp. CBMAI 2071, *Trichoderma atroviride* CBMAI 2073, and *Cladosporium cladosporioides* CBMAI 2075. Since these four strains could grow in a culture medium with cutin and polycaprolactone as the sole carbon sources, they could be categorized into the cutinase-producers.

##### 2.2.1.1 Yarrowia lipolytica

*Y. lipolytica* is a versatile, non-pathogenic yeast that has been granted GRAS (generally recognized as safe) status by the FDA, which has been extensively studied for its remarkable abilities to secrete proteins, undergo dimorphic transitions, and degrade hydrophobic substrates (Sun et al., 2021; Lu et al., 2021; Park and Ledesma-Amaro, 2023). Notably, its hydrophilic cell surface exhibits a strong affinity for hydrophobic materials, rendering it an ideal candidate for use in fixed bed biofilm reactors (Amaral et al., 2006; Botelho et al., 2020). Furthermore, *Y. lipolytica* is also celebrated for its lipase production, with enzymes distributed in extracellular, cell wall-bound, and intracellular fractions. Remarkably, even the ultrasound-treated cell debris has also been employed as a potent biocatalyst for the hydrolysis of fats (Fraga et al., 2018).

Recently, a wild strain of *Y. lipolytica*, designated IMUFRJ 50682, has been identified capable of biodegrading PET. When supplemented with 5 wt% watermelon peels, this strain could be induced to synthesize specific enzymes, which efficiently catalyze the enzymatic degradation of PET. This led to the production of a significantly higher concentration of TPA, peaking at 42.02 mmol L^−1^. Interestingly, TPA consumption was also found by *Y. lipolytica* IMUFRJ 50682 in YPD media with lower glucose content. Unfortunately, in these two studies the structures of the PET-degrading enzymes were not identified. Enzymatic extracts from this train, enhanced by supplementation with commercial cork and PET, demonstrated varied lipase and esterase activities. Optimal PET depolymerization and maximum TPA production were attainable when a blend consisting of 20 wt% PET, apple peels, and commercial cork was employed. Under these experimental conditions, the TPA concentration soared to 21.2 μmol L^−1^ in 7 days (Sales et al., 2021).

*Y. lipolytica* IMUFRJ 50682 was also reported to catalyze the conversion of EG into glycolic acid (GA) effectively (Carniel et al., 2023). It demonstrates remarkable tolerance to high EG concentrations (up to 2 M), and the GA production is decoupled from cell growth metabolism. Notably, when the agitation speed was increased to 450 rpm, the yield of GA could be increased by 1.12-fold after 72 h. However, the continuous accumulation of GA suggests that *Y. lipolytica* is incapable of fully metabolizing GA into carbon dioxide due to the incomplete oxidation pathway. While this characteristic presents a promising approach for the upcycling of PET to the more valuable GA, the specific metabolic pathways of the tested diols and the enzymes therein involved still require further elucidation.

##### 2.2.1.2 Microsphaeropsis arundinis

In a recent study, 100 fungal strains, sourced from environments rich in hydrocarbons, were screened for the potentials to break down post-consumer polymers (Malafatti-Picca et al., 2019). Out of the nine isolates exhibiting significant hydrolytic activity, two strains of *M. arundinis* (CBMAI 2109 and CBMAI 2110) were identified capable of degrading PET nanoparticles into TPA, with degradation rates of 2.0 ± 0.4% and 2.7 ± 0.9%. Furthermore, they were also effective in promoting weight loss of two different types of commercial PET bottles. Additionally, a significant positive correlation was observed between the enhanced ability to depolymerize PET and the increased activities of both lipase and esterase enzymes. It is important to note that BHET and MHET were also detected in concentrations exceeding 0.6 ppm among all PET by-products. After 14 days, the *M. arundinis* strain CBMAI 2109 showed a 0.5% reduction in PET mass, whereas strain CBMAI 2110 achieved a 0.16% reduction.

Although the use of whole-cell filamentous fungi for PET biodegradation was investigated, pinpointing the functional genes responsible for PET hydrolysis via genomic sequencing stands as an essential step.

##### 2.2.1.3 Candida antarctica

Though lipase B from *Candida antarctica* (CALB), known for the broad substrate-specificity, has been utilized in PET-degradation, it also stands out as a promising candidate for the development of efficient PETase with improved catalytic performance (Hwang et al., 2022; Kundys et al., 2018). This could be attributed to its capacity to degrade a broad spectrum of polyesters, combined with its thermal stability, catalytic versatility, and robust tolerance to organic solvents and ionic liquids.

Recently, CALB was applied for the hydrolysis of PET-derived diesters and PET trimers (Świderek et al., 2023). In this study, the key lies in unraveling the molecular mechanisms that govern catalytic performance. It was found that the pH level significantly influences the selectivity of CALB in the hydrolysis of BHET. Under acidic conditions (pH 5), CALB catalyzes the complete hydrolysis of BHET to TPA and EG, while under neutral to alkaline conditions (pH 7 and 9), it selectively hydrolyzes BHET to MHET and EG. This pH-dependent selectivity might be caused by the protonation state of the active site, which forms a hydrogen bond network facilitating substrate binding and hydrolyzing. Furthermore, both soluble and immobilized CALB can be effectively employed in pH-controlled bio-transformations, which enables selective production of either TPA or MHET.

Notably, the immobilized CALB (specifically Novozyme 435) has shown an outstanding ability to maintain its activity and preserve the product profile over eight consecutive reaction cycles, which underscores its suitability for industrial applications in the realm of polymer recycling and upcycling. This research carries significant implications for the high value added BHET, a product resulting from the organocatalytic depolymerization of PET. By offering a method to convert waste plastics into valuable chemicals, this work contributes to the development of a circular economy within the plastic industry, aligning with sustainable practices and resource conservation.

##### 2.2.1.4 Aspergillus

Cutinases, a class of esterases produced by diverse phytopathogenic organisms, are specialized in breaking down cutin, a biopolyester coating present on the surfaces of leaves and fruits (Sahu et al., 2023). These enzymes also exhibit potent hydrolytic capabilities, enabling the cleavage of ester bonds within aliphatic polyesters such as poly(ε-caprolactone) (PCL). Furthermore, cutinases are also capable of facilitating the hydrolysis of structurally more rigid aliphatic-aromatic polyesters, such as PET (Radley et al., 2023; Arnling Bååth et al., 2022). Examples include enzymes from *Fusarium solani pisi* (FsC), *Pseudozyma Antarctica*, *Aspergillus niger* (Nyyssölä et al., 2013), *Thielavia terrestris*, *Aspergillus oryzae* (AoC), *Thermobifida fusca*, and *Thermobifida alba*, to name just a few.

The cutinase from *Aspergillus oryzae* (AoC) (Liu et al., 2009), an enzyme capable of hydrolyzing ester bonds in polyesters like PET. AoC also showed a preference for longer chain esters and displayed higher catalytic efficiency for *p*NPB and *p*NPV substrates. The enzyme exhibited improved thermostability with a melting temperature (*T*_m_) of 59°C, which is higher than that of FsC (56°C). AoC was able to achieve nearly complete degradation (87%) of PCL films within 6 h, while FsC achieved only 30% degradation. Crystal structure analysis revealed, compared with FsC, two critical differences: an additional disulfide bond (Cys63–Cys76) and a topologically favored catalytic triad (Ser126, Asp181, and His194) with a continuous and deeper groove. It is hypothesized that these structural features can enhance hydrolytic activity, modify substrate specificity, and boost thermostability, especially during the degradation of synthetic polyesters like PCL.

The AoC was further engineered to improve the thermostability. For the most stable variant, an improvement of 6°C in the thermal unfolding temperature (*T*_m_) was achieved, along with a 10-fold increase in the half-life of enzyme activity at 60°C (Shirke et al., 2016). Surprisingly, the increased stability did not enhance the catalytic rate or the optimal reaction temperature.

##### 2.2.1.5 PET hydrolases from metagenome mining

Recently, a novel PET hydrolase, designated MG8, was discovered within the human saliva metagenome through an efficient bioinformatics approach (Eiamthong et al., 2022). The bioinformatics pipeline consisted of the following key steps: (1) extracting metagenomic data from the MGnify database through HMMER searches, using known PET-degrading enzymes as the query templates; (2) filtering candidate sequences by identifying the presence of essential catalytic triad residues; and (3) analyzing sequence similarity networks to identify potential PET hydrolase candidates from both marine and human microbiomes. Employing this methodology resulted in the discovery of seven potential PET hydrolases, designated as MG1 through MG7, which were derived from marine microbiomes. Additionally, three more candidates, namely MG8 to MG10, were sourced from human microbiome samples.

MG1, MG7, and MG8 demonstrated the highest catalytic activity during the hydrolysis of BHET. At 37°C, their activity levels reached up to 27% of that demonstrated by *Is*PETase. Furthermore, the BHET-hydrolyzing activity of these enzymes was enhanced with increased NaCl concentrations. Notably, MG8 showed outstanding PET-degrading activity. In comparison to *Is*PETase, it could more efficiently transform PET powder, resulting in a 26-fold increase in the production of MHET and a 3-fold increase in the production of TPA. At the optimal temperature of 55°C, MG8 generated approximately 83 times as much TPA as *Is*PETase. Additionally, when it came to degrading PET powder, MG8 outperformed several well-known mutants. Specifically, it was about 43 times more effective than *Is*PETase^W159H/S238F^, 21 times more efficient than ThermoPETase, 5 times more potent than DuraPETase, and 17–23 times superior to Tfu and HiC.

Subsequently, through genetic code expansion, the catalytic serine of MG8 was substituted with 2,3-diaminopropionic acid (DAP), and this substitution endows the enzyme with the ability to function as a covalent binder for PET bio-functionalization. The engineered MG8 (DAP) exhibited an approximately 20-fold increase in its ability to bind the enzyme to PET. This method is modular and can be extended to other catalytic enzymes, including protein-based biosensors, potentially broadening the applicability of the DAP system across various applications.

#### 2.2.2 Plant-associated cells

##### 2.2.2.1 Rhizobacteria

Employing plants for remediation purposes is truly a sustainable and eco-friendly technology, which harnesses the natural abilities of plants and their root-associated microorganisms to remove or neutralize harmful contaminants in various environments. Rhizoremediation stands out as an especially effective strategy for soil decontamination (Hussain et al., 2022), as it leverages the synergistic relationship between plants and rhizospheric microorganisms.

Recently, rhizobacteria capable of degrading PET plastic have been successfully isolated, and they can utilize PET as their sole carbon source in minimal salt media (Dhaka et al., 2023). Three prominent rhizospheric isolates were identified with significant PET degradation capabilities including *Priestia aryabhattai* VT 3.12, *Bacillus pseudomycoides* VT 3.15, and *Bacillus pumilus* VT 3.16. VT3.12, VT3.15, and VT3.16 were capable of biodegrading PET sheets, and over 28 days they led to weight losses of 40, 36, and 32%, respectively. When it came to the degradation of 300 μm PET powder, in just 18 days, they achieved degradation rates of 69, 66, and 64%, respectively. These findings showed the efficacy of pulverizing PET to accelerate its degradation, which can be ascribed to the increased surface area. In addition, VT3.12 displayed the highest degradation activity for PET powder, and the degradation rate was above 69%. Significant biodegradation was also confirmed by FTIR analysis, and the powdered samples exhibited greater bond cleavage and hydroxylation due to the larger surface area. This further highlights the potential of these bacterial strains in the environmental remediation of PET waste.

#### 2.2.3 Microalgae

To date, only a limited number of studies have documented the roles of wild algae in the degradation of plastics. Photosynthetic microorganisms (e.g., Algae) adhering to plastic surfaces are capable of secreting ligninolytic and exopolysaccharide enzymes, which are instrumental in depolymerizing the plastic material (Barone et al., 2024). For example, the biodegradation capabilities of two dominant cyanobacterial species, *Phormidium lucidum* and *Oscillatoria subbrevis*, isolated from polyethylene carry bags submerged in domestic sewage water has been investigated (Sarmah and Rout, 2018). Biodegradation of LDPE was remarkably evident as evidenced by changes in surface morphology, chemical structure, and weight loss. Also, after the treatment, the polyethylene structure displayed significant disruption, with large grooves visible on the surface. Enzymatic activity assays showed that in the cyanobacterial treatments laccase activity was higher than that of manganese peroxidase activity. Notably, it was also reported that the presence of PET would inhibit the growth of *Spirulina* sp., and the *Spirulina* sp.-mediated PET-biodegradation is more effective in saline systems, particularly at 7 ppt salinity (Hadiyanto et al., 2022).

Recently, the capacity of indigenous microalgae consortia to eliminate microplastics (MPs) from various wastewater treatment plants (WWTPs) has been explored (Afonso et al., 2024). Native microalgae consortia were used to eliminate microplastic, which were firstly pre-adapted to the specific conditions of the wastewater effluent. Results showed that the growth of microalgae in wastewater effluents could efficiently promote the removal of MPs. This not only helps in reducing contamination in the aquatic environment bu. generating valuable biomass. Substantial biomass production was achieved (maximum of 2.6 g L^−1^ dry weight), with successful removal of MPs ranging from 31 ± 25% to 82 ± 13%. Furthermore, the approach employed in the study requires only minimal adjustment of the culture conditions, which streamlines the integration of such systems into actual WWTP facilities. This strategy could mitigate environmental pollution while simultaneously producing valuable biomass, which can be harnessed for bioenergy generation.

A case in point is the microalga *Uronema africanum* isolated from waste plastic bags in a freshwater lake, which was capable of degrading low-density polyethylene (LDPE) sheets (Sanniyasi et al., 2021). This microalga was able to colonize the LDPE sheets indicating the potential for plastic degradation. *U. africanum* Borge is able to initiate the biodegradation of LDPE within 30 days of incubation, which was observed through the formation of green hair-like structures protruding from the LDPE sheets. Analysis of the LDPE surface showed that the sheets treated with *U. africanum* Borge exhibited erosions, abrasions, grooves, and ridges, which were absent on the control sheets. Chemical analysis of biodegradation revealed a significant difference in fatty acids and hydrocarbons between the control and the microalga-treated sample. Especially, FTIR analysis identified new functional groups (including carboxylic acids, esters, and alkenes), and the AFM analysis also confirmed the increased roughness and surface erosion on the LDPE sheets treated with *U. africanum* Borge. The radial disc-shaped attachment structures of the microalga *U. africanum* Borge was also discovered, with an average diameter of 20–30 μm. In addition, the ridges and grooves observed on the LDPE surface was similar to the filamentous morphology of the microalga, with a width of approximately 10–15 μm.

These findings were believed to provide a foundation for further exploration of biological degradation of plastics using green microalgae or other photosynthetic eukaryotes. Especially, it was suggested that through synthetic biology, the photosynthetic microorganisms (e.g., *P. tricornutum*) could indeed be engineered into a valuable platform for future biotechnological applications, particularly in the bioremediation of PET-contaminated seawater.

### 2.3 Archaea

Recently, it was reported that an archaeal promiscuous feruloyl esterase (PET46, RLI42440.1) was identified from Candidatus *Bathyarchaeota* archaeon B1_G2, which is capable of efficiently degrading the semi-crystalline PET powder (Perez-Garcia et al., 2023). PET46 displays the PET-degrading activity at a broad pH range of 5–8 and a broad temperature spectrum with the highest activity observed at 70°C. Notably, its catalytic activity could be markedly influenced, for example the addition of Zn^2+^ could result in an almost 2-fold increase. It was also observed the catalytic activity could be enhanced by two orders of magnitude with the addition of 10% acetone, as well as 5% dimethyl sulfoxide and N, N-dimethylformamide. Simultaneously, it displays increased activities in the hydrolysis of BHET and MHET.

**Figure 6:**
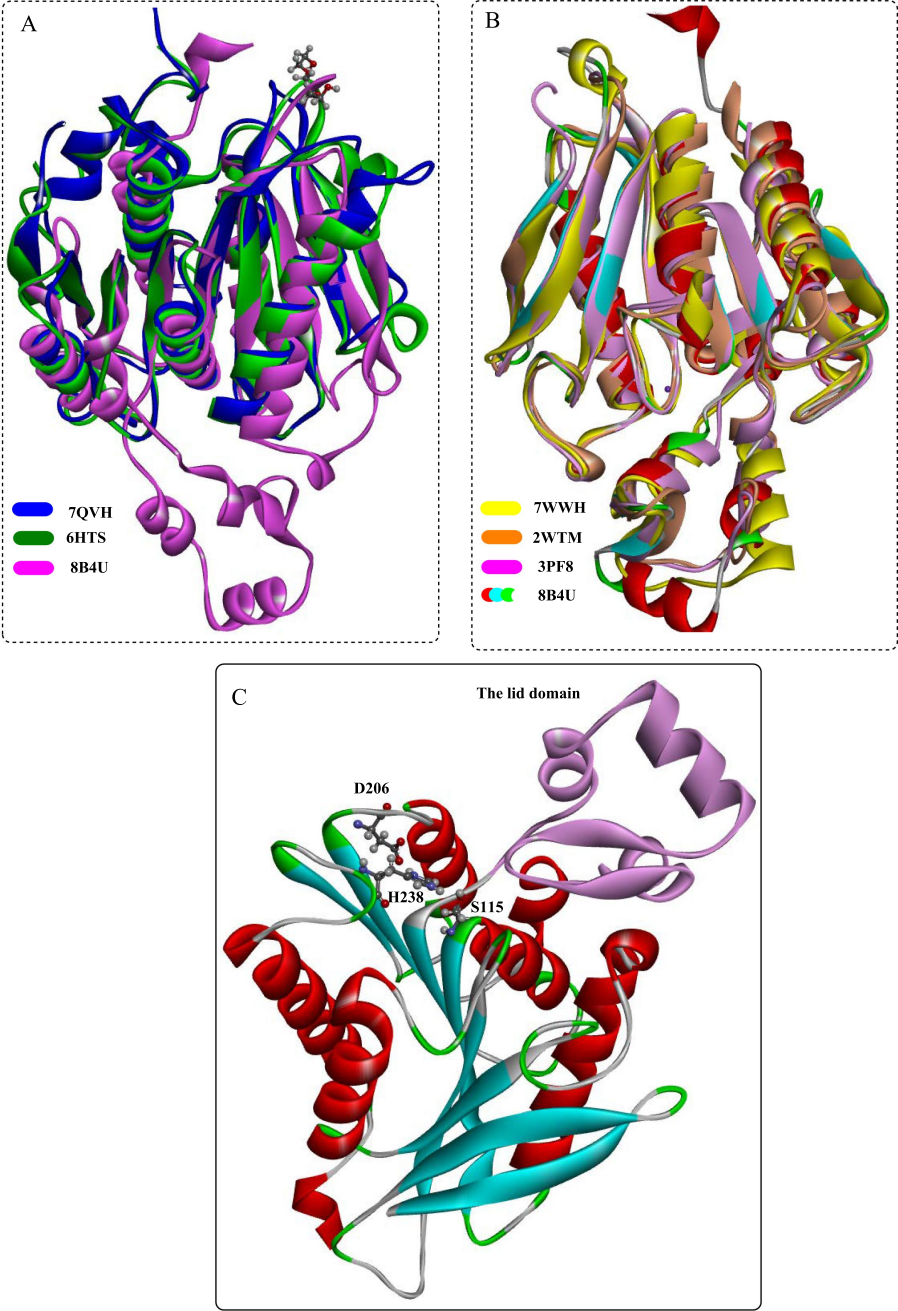
Structural analysis of the feruloyl esterase PET46. Structural alignment of PET46 with the bacterial PETases **(A)** and with the esterase **(B)**; **(C)** the crystal structural of PET46 and the catalytic triad.

PET46 exhibited a low degree of sequence similarity with bacterial PETases (Figure 6A), and the closest match (PETase Est1 from *Thermobifida alba*) had a sequence identity of only 41.6%. Structural analysis has indeed shown that PET46 also possesses the characteristic fold of the α/β-hydrolase superfamily, which is composed of eight β-strands connected by seven α-helices. The catalytic triad of PET46 is determined as Asp206, His238, and Ser115 based on the structural alignment. Furthermore, PET46 features a lid domain made up of three α-helices and two anti-parallel β-strands (Leu141-Val186, Figure 6C), which sets it apart from both *Is*PETase and LCC (Figure 4A). Another significant distinction of PET46 is the absence of the residue equivalent to Trp185 in *Is*PETase or Trp190 in LCC, which is critical for substrate recognition and facilitation of subsequent PET degradation (e.g., diacylation). This suggests that PET46 may employ a different substrate-binding pattern, potentially involving the lid domain in the formation of the aromatic clamp (Burgin et al., 2024). Mutation analysis revealed that Trp185 is essential for the formation of the aromatic clamp. Consequently, the absence of Trp185 in PET46 likely leads to a unique PET-binding mode for this enzyme.

Through structural comparison (Figure 6B), it was found that the best hit obtained with the highest structural similarity were the feruloyl esterases GthFAE (PDB ID:7WWH), Est1E (PDB ID: 2WTM) and the cinnamoyl esterase LJ0536 (PDB ID:3PF8). Among them, it was revealed that the only conserved residue in the lid is Phe178 of PET46 that is associated with the regulation of substrate binding. As a promiscuous feruloyl esterase, PET46 showed different levels of activity in hydrolyzing MHET, BHET, tri(2-hydroxyethyl) terephthalate (3PET), and PET polymers. It could convert more than 150 μM BHET into MHET and TPA in less than 30 min, and 50.99 μM TPA could be obtained after 1 h. Though the mutant PET46Δlid demonstrated the ability to catalyze the conversion of BHET into MHET (as high as 70.53 mM), the subsequent degradation of MHET was found to be inhibited. PET46 was capable of degrading all the 3PET into MHET and TPA within the first 3 h. In contrast, PET46Δlid could only catalyze half of the 3PET after 72 h, without the formation of BHET. Thus, it was estimated that the lid domain may play a role in facilitating efficient substrate binding and catalysis. In the degradation of semi-crystalline PET powder, the monomeric products released (including TPA, MHET, and BHET) exceeded 1.62 mM after 3 days at 60°C. Notably, over 99.1% of these products were identified as TPA.

### 2.4 Insect gut symbionts

#### 2.4.1 Tenebrio molitor

**Figure 7:**
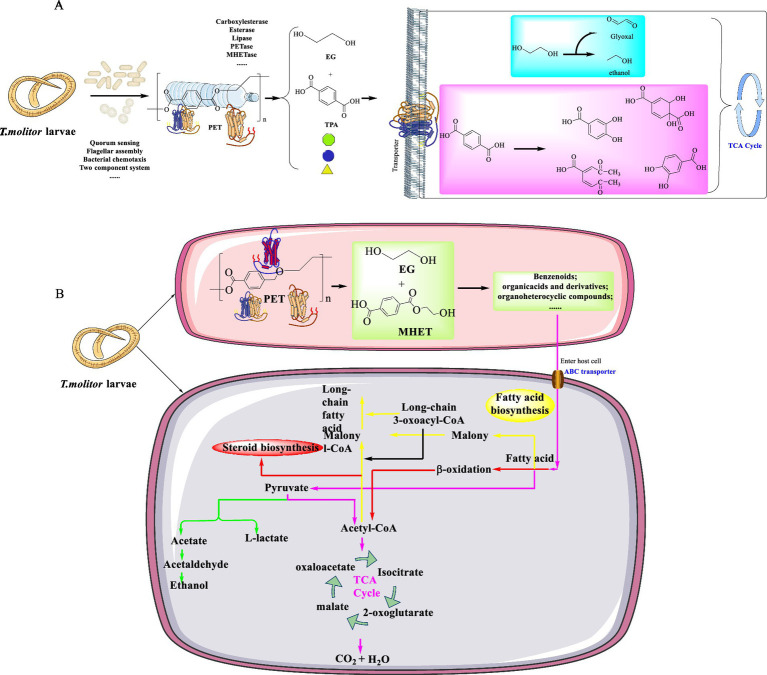
The inferred pathways for PET degradation contributed by the gut microbiomes of *T. molitor* larvae **(A)** and the host *T. molitor* larvae itself **(B)**.

*T. molitor* was discovered to possess the ability of efficiently degrading two distinct varieties of commercial PET resins with varying molecular weights and degrees of crystallinity (He et al., 2023). This digestive process led to a significant reduction in mass with the specific removal rate more than 136 mg PET 100 larvae^−1^ d^−1^ and 152 mg PET 100 larvae^−1^ d^−1^ after 36 days. The average weights of larvae fed on various diets highlights its adaptability in utilizing a range of substrates to meet their metabolic requirements, and this demonstrates their capacity to extract energy from the digestion or biodegradation of PET. However, this capability does not ensure the provision of adequate nutrition or essential minerals required for the sustained growth and development. This indicates that plastics, including PET, cannot serve as a sustainable sole diet for sustaining their life processes in the long run. Over a 36-day cultivation, the specific consumption rates (SPCRs) of *T. molitor* larvae on PET-2 initially surpassed those on PET-1. Then they experienced a decline by the 12th and 16th day, and subsequently increased again, which indicates an adaptive response to the PET-based diet. This adaptation, potentially facilitated by changes in gut microbiota, was also evidenced by higher average SPCRs for both PET types compared to previous studies on different plastics. This finding further highlights the significant capacity of *T. molitor* to consume PET. The consumption of a PET diet by the larvae would significantly alter their gut microbiota (Figure 7A), with a notable increase in the relative abundance of such as *Spiroplasmataceae*, *Enterococcaceae*, and *Dysgonomonadaceae*. Among the dominant genera, *Enterococcus* sp. and *Spiroplasma* sp. were known for the plastic-degrading capabilities. Furthermore, PET-degradation by larvae was also evidenced by the conversion of PET into smaller organic molecules like formic and acetic acids, which were facilitated by the over-expressed enzymes (e.g., EC 1.97.1.4, EC1.2.1.2, EC 2.3.1.54, and EC2.8.3.8). Especially, a remarkable increase in the expression of oxidases and hydrolases was highlighted, which are likely involved in the oxidation and breakdown of PET into smaller organic molecules. These products would potentially enter the TCA cycle for further metabolism, which was confirmed by the up-regulation of related enzymes. In the group fed with PET, the upregulated genes usually capable of encoding the methyl-accepting chemotaxis proteins, tight adherence-related proteins, and flagellin-related proteins. This implies that these proteins might play a crucial role in guiding chemotaxis toward aromatic compounds, which could bolster bacterial adhesion for efficient biodegradation, and facilitate electron transport for metabolic processes. These functions are vital for the adaptation of gut microbes to the PET diet and their abilities to effectively degrade PET. In addition, PET-degradation might be also potentially enhanced by electron transfer via conductive flagella, where the nitrogen-fixing bacteria plays a supplementary role in nitrogen-limited conditions.

Metabolite analysis also uncovered notable changes in the gut microbiota of *T. molitor* larvae fed with PET, and this indicates a sophisticated microbial response to PET biodegradation and the engagement of diverse metabolic pathways in this process. Furthermore, it was proposed that both aerobic and facultative anaerobic gut bacteria in *T. molitor* larvae contribute to PET biodegradation. The integration of metabolic products into the TCA cycle, coupled with nitrogen-fixing microbes alleviating nitrogen deficiency, suggests that plastic diets might significantly alter the gut microbiota and the physiological functions of the host. This intricate interplay highlights the adaptability of the gut microbiome to the presence of plastics and the role in the abilities to process such materials.

Recently, the biodegradation of commercial PET (molecular weight 29.43 kDa) catalyzed by *T. molitor* was confirmed by the δ^13^C signature (He et al., 2024). The signature was increased from −27.50 ± 0.18‰ to −26.05 ± 0.16‰, which clearly highlights the *T. molitor*-mediated PET-biodegradation. This strategy represents a much more efficient method with higher degradation rates compared with the bacterial-facilitated PET-biodegradation. Furthermore, the degradation of high-crystallinity commercial PET degradation facilitated by the larvae of *T. molitor* was significantly faster than that catalyzed by *I. sakaiensis* 201-F6, which led to a remarkable 99-fold and 266-fold increase after 4 days and 36 days, respectively. Even when the gut microbes were suppressed by antibiotics, PET depolymerization still proceeded independently. Confirmed by the metagenome sequencing, it was shown that the gut microbiota of *T. molitor* larvae are capable of expressing various enzymes to modify PET surfaces (e.g., esterase/lipase, carboxylesterase), metabolize the released products (carbohydrate esterase enzymes), and provide essential nutrients through nitrogen fixation. Based on these findings, it was suggested that the degradation process mainly involves three main steps: initial physical breakdown, enzymatic depolymerization, and intracellular metabolism (Figure 7B). Finally, it would lead to the mineralization of intermediates into CO_2_ and H_2_O, with some carbons assimilated into biomass. This process is more efficient than reported microbial *in vitro* degradation or environmental degradation, with a half-life of PET less than 4 h, which is believed to be a promising approach to PET waste management.

#### 2.4.2 Hymenoptera gut-derived microbes

Recently, two bacterial strains (HY-74 strain, HY-75 strain) from *Hymenoptera* were identified with high PET-degradation potential, which are closely related to *Xanthomonas sontii* (99.92% 16S rRNA sequence similarity with strain HY-74) and *Bacillus siamensis* (99.57% 16S rRNA sequence similarity with strain HY-75). These two strains demonstrated the ability to produce extracellular enzymes (e.g., protease, PCL-degrading enzyme, and lipase), which was also evidenced by the formation of transparent halo zones and calcium complexes.

In the degradation of PET film, after 6 weeks, both the HY-74 and HY-75 strains demonstrated significant degradation activities, with weight losses of 1.57% ± 0.21% for HY-74 and 1.42% ± 0.46% for HY-75. These changes were accompanied by distinct morphological alterations that were observed under scanning electron microscopy (SEM) and subsequently confirmed by Fourier-transform infrared spectroscopy (FTIR) analysis. Regarding the degradation of PET powder, TPA was detected in the degradation products after just 4 weeks, with both strains demonstrating the ability to catalyze its formation. However, MHET and BHET were not detected, which is estimated that they were further hydrolyzed to TPA. This study underscores the promise of bacteria derived from insects in breaking down PET, and emphasizes the needs for further research into the practical applications to help address the worldwide issue of plastic pollution.

### 2.5 Aquatic microorganisms

#### 2.5.1 The deep-sea bacteria

Recently, the deep-sea bacteria collected from the eastern central Pacific Ocean were investigated for the PET-degrading capability (Zhao et al., 2023). From a total of 81 different bacterial strains, four bacteria were confirmed to possess PET-degrading ability including *Marinobacter sediminum* BC31_3_A1, *Marinobacter gudaonensis* BC06_2_A6, *Thalassospira xiamenensis* BC02_2_A1 and *Nocardioides marinus* BC14_2_R3. Among them, BC02_2_A1 showed a much faster growth rate than others with higher biomass obtained. After 30-day incubation, the plastic weight loss catalyzed by BC31_3_A1, BC06_2_A6, BC02_2_A1 and were 1.3 ± 0.2%, 1.2 ± 0.07%, 1.3 ± 0.2% and 1.8 ± 0.2%, respectively. These results corresponds to the reported degradation rate (1–2%) of granular PET particles catalyzed by *Bacillus thuringiensis* C15, *Diphtheria* strain PFYN01 and *Pseudomonas* strain SWI36 at 30°C for 6 weeks (Zhao et al., 2023). In addition, the key degradation products were determined to be MHET and TPA monomers. Though this study offered limited insights into the mechanisms underlying PET degradation, it highlights the importance of the widespread presence and remarkable diversity of PET-degrading bacterial consortia in the deep ocean environments.

In another study, pelagic sediments from 19 diverse Pacific Ocean sites were screened for microorganisms with the ability to degrade PET (Liu et al., 2024). This investigation resulted in the identification of 10 microbial samples demonstrating the capability to utilize PET as their sole carbon and energy source, which could achieve PET removal rates ranging from 1.8% (consortium BC11) to 16.2% (BC14) after 2 months. Microbial diversity analysis indicated that *Alcanivorax* and *Pseudomonas* were dominant in all the PET-degrading consortia, with additional taxa including *Thalassospira*, *Nitratireductor*, *Nocardioides*, *Muricauda*, and *Owenweeksia* showing PET degradation potential. Notably, the findings demonstrate that the pure cultures of *Alcanivorax* sp. A02-7 and *Pseudomonas* sp. A09-2 exhibited biofilm development on PET substrates. UPLC-MS analysis revealed that *Alcanivorax* sp. A02-7 and *Pseudomonas* sp. A09-2 were capable of bio-transforming PET into MHET within 4 weeks, without the release of any TPA or EG. Remarkably, this transformation was successfully achieved under simulated deep-sea conditions, maintaining metabolic activity despite the combined stressors of high hydrostatic pressure and low temperature.

#### 2.5.2 Shallow water microorganisms

In a recent study, it highlighted the PET degradation potential of indigenous microbial strains isolated from plastic waste accumulation sites along the Kaveri River basin, demonstrating their natural capability to degrade PET films (Maheswaran et al., 2023). It identified multiple microbial strains demonstrating PET film degradation capability at 37°C. Particularly noteworthy was the microbial consortium comprising *S. aurantiaca*, *B. subtilis*, *A. flavus*, and *A. niger*, which achieved a significant PET film weight reduction of 28.78% in Bushnell-Hass medium. SEM analysis further revealed the substantial surface erosion and structural modifications of the PET films. Especially, the observed extensive biofilm formation provided strong evidence of their remarkable proficiency in both surface colonization and subsequent PET degradation, which highlights an adaptive mechanism for enhancing polymer breakdown efficiency. Therefore, it was concluded that the consortium could be used to treat PET waste without posing health or environmental risks and has the potential to degrade PET at plastic-contaminated sites.

These studies illuminate the complex interactions between aquatic microorganisms and plastic materials in aquatic environments. As highlighted (Di Pippo et al., 2023; Pires et al., 2024), it is imperative to focus on understanding the dynamics of the plastisphere, which plays a pivotal role in mitigating the environmental consequences of plastic pollution especially the marine plastic pollution.

## 3 Discussion and conclusion

The microbial biodegradation of PET has emerged as a rapidly evolving research frontier, and the recent groundbreaking studies have also provided unprecedented insights into both the enzymatic mechanisms and biotechnological potential of this biological process for plastic waste management. Therefore, harnessing the potential of microorganisms for the biodegradation of PET is of paramount importance, which would offer a sustainable alternative for breaking down PET into simpler, less harmful components, and contributing to a circular economy. Despite significant advancements in microbial-mediated PET biodegradation research, several critical challenges persist, particularly in the areas of degradation efficiency optimization, enzymatic pathway engineering, and practical implementation strategies, which require concerted research efforts to bridge the gap between laboratory-scale investigation and practical applications.

### 3.1 Challenges in microbial PET breakdown

#### 3.1.1 Scalability challenges in microbial PET breakdown

Scaling up microbial PET degradation from laboratory-scale experiments to industrial-scale processes is a complex task. In the practical application of microbe-mediated PET degradation, maintaining consistent growth conditions for PET-degrading microorganisms becomes increasingly difficult. For example, ensuring homogeneous distribution of nutrients throughout the large volume of the bioreactor is a challenge. Oxygen transfer can also be a limiting factor in aerobic systems, as the demand for oxygen increases with the scale of the operation. This can lead to sub-optimal growth of aerobic PET-degrading microorganisms, reducing the overall efficiency of the degradation process. Therefore, specialized equipment is usually required to control parameters such as temperature, pH, and agitation, which further adds to the cost. To address these scalability issues, continuous-flow bioreactors would provide a more stable environment for microbial growth and continuous production of degradation products. In addition, immobilized cell systems are capable of improving the separation of cells from the reaction mixture, facilitating easier reuse of the cells and potentially reducing the cost of the process.

#### 3.1.2 Enzyme stability

Enzyme stability is another crucial factor in the practical application of microbial PET degradation. PET-depolymerizing enzymes are usually exposed to harsh environmental conditions (high temperatures [near the *T_g_*], extreme pH, a specific ionic organic solvents environment, nature of the PET substrate) during the degradation process. To surmount these challenges, protein engineering and enzyme immobilization techniques have been proven to be effective in enhancing the performance of PET-degrading enzymes, such as improving their catalytic activity, thermostability, and reusability.

#### 3.1.3 Economic hurdles in microbial PET breakdown

The cost of microbial PET breakdown is also a major concern that needs to be addressed for its widespread adoption. This mainly includes the cost of microbial growth media, and the production (e.g., the fermentation process) and purification of PET-degrading enzymes. To reduce the cost of microbial growth, exploring alternative, inexpensive growth substrates is a promising strategy, for example use of agricultural waste or industrial by-products as growth substrates. Moreover, improving the efficiency of enzyme production through protein engineering or metabolic engineering and optimization of fermentation conditions could also help lower the overall cost of the process.

#### 3.1.4 Environmental impacts

While microbial PET degradation is generally considered an environmentally friendly approach, there are potential environmental implications that need to be carefully evaluated, such as the release of unpredictable intermediate products. Some of these intermediate products could be toxic to certain organisms, potentially disrupting the ecological balance. For example, TPA and EG, the main intermediate products of PET degradation, might display unforeseen effects on the microorganisms in the surrounding environment. In light of this, conducting comprehensive environmental impact assessments and optimizing the metabolic pathways of PET-degrading microorganisms are crucial for the practical application of PET bio-degradation. This could be achieved through metabolic engineering or engineering through synthetic biology, such as knocking out genes involved in the production of toxic intermediates, or by engineering the chassis cell with more favorable metabolic profiles.

### 3.2 Future research directions

The compelling need to leverage the capabilities of microorganisms in the biodegradation of PET is both multifaceted and urgent. Unearthing and identifying potential microbial degraders is the foundational and critical first step in this significant endeavor. Building upon this, there is a pressing need to focus on the following areas.

#### 3.2.1 Incorporating technologies into current recycling systems

Integrating microbial technologies into current recycling systems is a promising direction for improving the efficiency of PET recycling. Development of enhanced microbial chassis is a strategic approach that holds significant promise in addressing the global issue of plastic pollution, particularly with respect to PET waste. Genetic modification of microorganisms, known as microbial chassis (including bacteria, fungi, and marine microalgae), allows for the introduction of new traits that enhance their ability to degrade PET. Especially, the use of photosynthetic microalgae as microbial chassis represents an innovative approach. The microalgae could be rationally engineered to produce and secrete PET-degrading enzymes like PETase, which function under mesophilic marine conditions, which indicates their potentials for PET degradation in natural environments. On the other hand, the microbial chassis engineered would be capable of not only degrading PET but also utilizing it as a carbon source, which could lead to the complete metabolism of PET into harmless byproducts such as carbon dioxide and water. Furthermore, the enhanced microbial chassis would also play a pivotal role in realizing the circular economy of PET waste by converting waste into valuable chemicals or new PET materials, thus reducing the reliance on fossil feedstocks and the environmental impact of plastic waste.

#### 3.2.2 Construction of artificial microbial consortia for PET degradation

Artificial microbial consortia can improve the degradation rates of PET by alleviating the inhibition caused by its breakdown products (e.g., TPA, EG). Incorporation of different species targeting specific degradation products could enhance the overall efficiency of the biodegradation process. On the one hand, it allows for metabolic cross-feeding or the production of metabolites that induce co-metabolic degradation. On the other hand, it could reduce the metabolic burden on each strain and increase the tolerance of the microbial community to harsh environments. Therefore, compared to pure cultures, artificial microbial consortia offer advantages in stability and efficiency within the growth environment, which could provide a suitable catalytic environment for each enzyme required by the biodegradation pathway. Beyond just degradation, the research also opens avenues for converting PET into high-value chemicals. The consortium not only degrades PET but also addresses the conversion of degradation products into usable materials, thus adding economic value to the process.

#### 3.2.3 Development of multienzyme systems

The development of multienzyme systems for the depolymerization of plastic waste is a significant research area. For instance, the dual enzyme system of PETase and MHETase in *Ideonella sakaiensis* strain has shown the ability to utilize crystalline polyester substrates, and there is potential to improve these enzymes through genetic engineering. In addition, it is well known that one of the challenges in PET degradation is product inhibition, where the accumulation of intermediates like MHET inhibits the activity of PETase. The use of multienzyme systems can mitigate this by rapidly converting these intermediates into end products, thus enhancing the overall degradation efficiency. Multienzyme systems could also be designed to improve the stability and robustness of enzymes in inhospitable environments. For instance, a customized self-assembled synergistic biocatalyst (MC@CaZn-MOF) was developed to promote the two-step depolymerization process, showing better adhesion to the PET surface and desirable durability.

#### 3.2.4 Improving enzyme engineering

To enhance the performance of PET-degrading enzymes, advanced protein engineering techniques should be further explored. This could further address the challenges of enhancing the catalytic performance of these enzymes at near-ambient temperatures and improving the efficiency of PET degradation. This includes the use of directed evolution and rational design to improve enzyme performance. Through biotechnology, it has been successful in creating mutants with enhanced hydrolysis activity and stability. Furthermore, it has been shown that the substrate binding step has been proven to be particularly important in determining the catalytic efficiency of the enzyme. The combination of PETase with adsorption modules, such as hydrophobic binding modules, can greatly facilitate enzyme binding to the substrate and enhance PET degradation efficiency. (5) Smart technologies and bioinformatics are revolutionizing the biodegradation of PET through the application of computational biology, machine learning, and advanced bioinformatics tools. These cutting-edge technologies are instrumental in the field of PET degradation, particularly in the design of synthetic metabolic pathways and the prediction of enzyme-substrate interactions, which are crucial for enhancing the efficiency and specificity of PET breakdown processes. For example, structural bioinformatics-based protein engineering could be used to optimize the substrate binding site of PET-degrading enzymes like PETase, leading to variants with higher enzymatic activity at mild temperatures or under industrially relevant conditions. The integration of bioinformatics tools (e.g., machine learning and quantum mechanics/molecular mechanics) enables the prediction of enzyme-substrate interactions and the improvement of reaction conditions for PET degradation. In addition, these tools are also capable of simulating and analyzing the behavior of enzymes at the molecular level, leading to a better understanding of the degradation mechanisms and the development of more effective biodegradation strategies. The development of tandem chemical-biological approaches could leverage the advantages of both chemical and biological depolymerization processes, which aims to improve the overall efficiency of PET recycling and upcycling. This involves a chemical pretreatment, such as glycolysis, followed by enzymatic degradation using enzymes like ΔBsEst and ΔChryBHETase to yield pure TPA from intermediate BHET.

The biodegradation of PET by microorganisms is a promising approach to address the environmental challenges posed by this plastic. Research has identified key microbial species and enzymatic pathways involved in PET degradation, and innovative strategies are being explored to enhance the process. The use of microbial consortia and engineered enzymes holds potential for the management of PET waste, contributing to a more sustainable circular economy.

## References

[ref1] Abel S. M. Wu F. Primpke S. Gerdts G. Brandt A. (2023). Journey to the deep: plastic pollution in the hadal of deep-sea trenches. Environ. Pollut. 333:122078. doi: 10.1016/j.envpol.2023.122078, PMID: 37379878

[ref2] Afonso V. Borges R. Rodrigues B. Barros R. João Bebianno M. Raposo S. (2024). Are native microalgae consortia able to remove microplastics from wastewater effluents? Environ. Pollut. 349:123931. doi: 10.1016/j.envpol.2024.123931, PMID: 38582186

[ref3] Ahmaditabatabaei S. Kyazze G. Iqbal H. M. N. Keshavarz T. (2021). Fungal enzymes as catalytic tools for polyethylene terephthalate (PET) degradation. J. Fungi (Basel) 7:931. doi: 10.3390/jof7110931, PMID: 34829219 PMC8625934

[ref4] Amalia L. Chang C. Y. Wang S. S. Yeh Y. C. Tsai S. L. (2023). Recent advances in the biological depolymerization and upcycling of polyethylene terephthalate. Curr. Opin. Biotechnol. 85:103053. doi: 10.1016/j.copbio.2023.103053, PMID: 38128200

[ref5] Amaral P. F. Lehocky M. Barros-Timmons A. M. Rocha-Leão M. H. Coelho M. A. Coutinho J. A. (2006). Cell surface characterization of Yarrowia lipolytica IMUFRJ 50682. Yeast 23, 867–877. doi: 10.1002/yea.1405, PMID: 17001615

[ref6] Amobonye A. Bhagwat P. Singh S. Pillai S. (2021). Plastic biodegradation: frontline microbes and their enzymes. Sci. Total Environ. 759:143536. doi: 10.1016/j.scitotenv.2020.143536, PMID: 33190901

[ref7] Arnling Bååth J. Novy V. Carneiro L. V. Guebitz G. M. Olsson L. Westh P.. (2022). Structure-function analysis of two closely related cutinases from *Thermobifida cellulosilytica*. Biotechnol. Bioeng. 119, 470–481. doi: 10.1002/bit.27984, PMID: 34755331 PMC9299132

[ref8] Arpia A. A. Chen W. H. Ubando A. T. Naqvi S. R. Culaba A. B. (2021). Microplastic degradation as a sustainable concurrent approach for producing biofuel and obliterating hazardous environmental effects: a state-of-the-art review. J. Hazard. Mater. 418:126381. doi: 10.1016/j.jhazmat.2021.126381, PMID: 34329008

[ref9] Austin H. P. Allen M. D. Donohoe B. S. Rorrer N. A. Kearns F. L. Silveira R. L.. (2018). Characterization and engineering of a plastic-degrading aromatic polyesterase. Proc. Natl. Acad. Sci. USA 115, E4350–e4357. doi: 10.1073/pnas.1718804115, PMID: 29666242 PMC5948967

[ref10] Barone G. D. Rodríguez-Seijo A. Parati M. Johnston B. Erdem E. Cernava T.. (2024). Harnessing photosynthetic microorganisms for enhanced bioremediation of microplastics: a comprehensive review. Environ. Sci. Ecotechnol. 20:100407. doi: 10.1016/j.ese.2024.100407, PMID: 38544950 PMC10965471

[ref11] Botelho A. Penha A. Fraga J. Barros-Timmons A. Coelho M. A. Lehocky M.. (2020). Yarrowia lipolytica adhesion and immobilization onto residual plastics. Polymers (Basel) 12:649. doi: 10.3390/polym12030649, PMID: 32178341 PMC7182813

[ref12] Burgin T. Pollard B. C. Knott B. C. Mayes H. B. Crowley M. F. McGeehan J. E.. (2024). The reaction mechanism of the Ideonella sakaiensis PETase enzyme. Commun. Chem. 7:65. doi: 10.1038/s42004-024-01154-x, PMID: 38538850 PMC10973377

[ref13] Carniel A. Santos A. G. Júnior Chinelatto L. S. Castro A. M. Coelho M. A. Z. (2023). Biotransformation of ethylene glycol to glycolic acid by Yarrowia lipolytica: a route for poly(ethylene terephthalate) (PET) upcycling. Biotechnol. J. 18:e2200521. doi: 10.1002/biot.202200521, PMID: 36896762

[ref001] de Souza CP. Ribeiro BD. Zarur Coelho MA Almeida RV Nicaud JM. (2020). Construction of wild-type Yarrowia lipolytica IMUFRJ 50682 auxotrophic mutants using dual CRISPR/Cas9 strategy for novel biotechnological approaches. Enzyme Microb Technol. 140:109621. doi: 10.1016/j.enzmictec.2020.109621, PMID: 32912681

[ref14] Delacuvellerie A. Cyriaque V. Gobert S. Benali S. Wattiez R. (2019). The plastisphere in marine ecosystem hosts potential specific microbial degraders including *Alcanivorax borkumensis* as a key player for the low-density polyethylene degradation. J. Hazard. Mater. 380:120899. doi: 10.1016/j.jhazmat.2019.120899, PMID: 31326835

[ref15] Dhaka V. Singh S. Ramamurthy P. C. Samuel J. Naik T. S. S. K. Khasnabis S.. (2023). Biological degradation of polyethylene terephthalate by rhizobacteria. Environ. Sci. Pollut. Res. Int. 30, 116488–116497. doi: 10.1007/s11356-022-20324-9, PMID: 35460002

[ref16] Di Pippo F. Bocci V. Amalfitano S. Crognale S. Levantesi C. Pietrelli L.. (2023). Microbial colonization patterns and biodegradation of petrochemical and biodegradable plastics in lake waters: insights from a field experiment. Front. Microbiol. 14:1290441. doi: 10.3389/fmicb.2023.1290441, PMID: 38125574 PMC10731271

[ref17] Diao J. Hu Y. Tian Y. Carr R. Moon T. S. (2023). Upcycling of poly(ethylene terephthalate) to produce high-value bio-products. Cell Rep. 42:111908. doi: 10.1016/j.celrep.2022.111908, PMID: 36640302

[ref18] Dissanayake L. Jayakody L. N. (2021). Engineering microbes to bio-upcycle polyethylene terephthalate. Front. Bioeng. Biotechnol. 9:656465. doi: 10.3389/fbioe.2021.656465, PMID: 34124018 PMC8193722

[ref19] Eiamthong B. Meesawat P. Wongsatit T. Jitdee J. Sangsri R. Patchsung M.. (2022). Discovery and genetic code expansion of a polyethylene terephthalate (PET) hydrolase from the human saliva metagenome for the degradation and bio-functionalization of PET. Angew. Chem. Int. Ed. Engl. 61:e202203061. doi: 10.1002/anie.202203061, PMID: 35656865 PMC7613822

[ref20] Fraga J. L. Penha A. C. B. da S Pereira A. Silva K. A. Akil E. Torres A. G.. (2018). Use of Yarrowia lipolytica lipase immobilized in cell debris for the production of lipolyzed milk fat (LMF). Int. J. Mol. Sci. 19:3413. doi: 10.3390/ijms19113413, PMID: 30384435 PMC6274823

[ref21] Gao Z. Ma B. Chen S. Tian J. Zhao C. (2022). Converting waste PET plastics into automobile fuels and antifreeze components. Nat. Commun. 13:3343. doi: 10.1038/s41467-022-31078-w, PMID: 35688837 PMC9187643

[ref22] Gao R. Pan H. Kai L. Han K. Lian J. (2022). Microbial degradation and valorization of poly(ethylene terephthalate) (PET) monomers. World J. Microbiol. Biotechnol. 38:89. doi: 10.1007/s11274-022-03270-z, PMID: 35426614

[ref23] Guo W. Duan J. Shi Z. Yu X. Shao Z. (2023). Biodegradation of PET by the membrane-anchored PET esterase from the marine bacterium *Rhodococcus pyridinivorans* P23. Commun. Biol. 6:1090. doi: 10.1038/s42003-023-05470-1, PMID: 37891241 PMC10611731

[ref24] Hachisuka S. I. Chong J. F. Fujiwara T. Takayama A. Kawakami Y. Yoshida S. (2022). Ethylene glycol metabolism in the poly(ethylene terephthalate)-degrading bacterium Ideonella sakaiensis. Appl. Microbiol. Biotechnol. 106, 7867–7878. doi: 10.1007/s00253-022-12244-y, PMID: 36289066

[ref25] Hachisuka S. I. Nishii T. Yoshida S. (2021). Development of a targeted gene disruption system in the poly(ethylene terephthalate)-degrading bacterium Ideonella sakaiensis and its applications to PETase and MHETase genes. Appl. Environ. Microbiol. 87:e0002021. doi: 10.1128/aem.00020-21, PMID: 34260304 PMC8388835

[ref26] Hadiyanto H. Muslihuddin M. Khoironi A. Pratiwi W. Z. Fadlilah M. N. Muhammad F.. (2022). The effect of salinity on the interaction between microplastic polyethylene terephthalate (PET) and microalgae Spirulina sp. Environ. Sci. Pollut. Res. Int. 29, 7877–7887. doi: 10.1007/s11356-021-16286-z, PMID: 34480706

[ref27] Han Z. Nina M. R. H. Zhang X. Huang H. Fan D. Bai Y. (2024). Discovery and characterization of two novel polyethylene terephthalate hydrolases: one from a bacterium identified in human feces and one from the Streptomyces genus. J. Hazard. Mater. 472:134532. doi: 10.1016/j.jhazmat.2024.134532, PMID: 38749251

[ref28] He L. Yang S. S. Ding J. Chen C. X. Yang F. He Z. L.. (2024). Biodegradation of polyethylene terephthalate by *Tenebrio molitor*: insights for polymer chain size, gut metabolome and host genes. J. Hazard. Mater. 465:133446. doi: 10.1016/j.jhazmat.2024.133446, PMID: 38219578

[ref29] He L. Yang S. S. Ding J. He Z. L. Pang J. W. Xing D. F.. (2023). Responses of gut microbiomes to commercial polyester polymer biodegradation in *Tenebrio molitor* larvae. J. Hazard. Mater. 457:131759. doi: 10.1016/j.jhazmat.2023.131759, PMID: 37276692

[ref30] Hussain A. Rehman F. Rafeeq H. Waqas M. Asghar A. Afsheen N.. (2022). In-situ, ex-situ, and nano-remediation strategies to treat polluted soil, water, and air - a review. Chemosphere 289:133252. doi: 10.1016/j.chemosphere.2021.133252, PMID: 34902385

[ref31] Hwang D. H. Lee M. E. Cho B. H. Oh J. W. You S. K. Ko Y. J.. (2022). Enhanced biodegradation of waste poly(ethylene terephthalate) using a reinforced plastic degrading enzyme complex. Sci. Total Environ. 842:156890. doi: 10.1016/j.scitotenv.2022.156890, PMID: 35753492

[ref32] Jabloune R. Khalil M. Ben Moussa I. E. Simao-Beaunoir A. M. Lerat S. Brzezinski R.. (2020). Enzymatic degradation of p-nitrophenyl esters, polyethylene terephthalate, cutin, and suberin by sub1, a suberinase encoded by the plant pathogen Streptomyces scabies. Microbes Environ. 35:19086. doi: 10.1264/jsme2.ME19086, PMID: 32101840 PMC7104285

[ref33] Jiang W. Sun J. Dong W. Zhou J. Jiang Y. Zhang W.. (2023). Characterization of a novel esterase and construction of a Rhodococcus-Burkholderia consortium capable of catabolism bis (2-hydroxyethyl) terephthalate. Environ. Res. 238:117240. doi: 10.1016/j.envres.2023.117240, PMID: 37783328

[ref34] Klauer R. R. Hansen D. A. Wu D. Monteiro L. M. O. Solomon K. V. Blenner M. A. (2024). Biological upcycling of plastics waste. Annu. Rev. Chem. Biomol. Eng. 15, 315–342. doi: 10.1146/annurev-chembioeng-100522-115850, PMID: 38621232 PMC11575423

[ref35] Knott B. C. Erickson E. Allen M. D. Gado J. E. Graham R. Kearns F. L.. (2020). Characterization and engineering of a two-enzyme system for plastics depolymerization. Proc. Natl. Acad. Sci. USA 117, 25476–25485. doi: 10.1073/pnas.2006753117, PMID: 32989159 PMC7568301

[ref36] Kundys A. Biaecka-Florjańczyk E. Fabiszewska A. Maajowicz J. (2018). Candida antarctica lipase B as catalyst for cyclic esters synthesis, their polymerization and degradation of aliphatic polyesters. J. Polym. Environ. 26, 396–407. doi: 10.1007/s10924-017-0945-1

[ref37] Lee M. Kim H. Ryu H. S. Moon J. Khant N. A. Yu C.. (2022). Review on invasion of microplastic in our ecosystem and implications. Sci. Prog. 105:368504221140766. doi: 10.1177/00368504221140766, PMID: 36426552 PMC10306144

[ref38] Li X. Wu H. Gong J. Li Q. Li Z. Zhang J. (2023). Improvement of biodegradation of PET microplastics with whole-cell biocatalyst by interface activation reinforcement. Environ. Technol. 44, 3121–3130. doi: 10.1080/09593330.2022.2052359, PMID: 35293270

[ref39] Liu Z. Gosser Y. Baker P. J. Ravee Y. Lu Z. Alemu G.. (2009). Structural and functional studies of aspergillus oryzae cutinase: enhanced thermostability and hydrolytic activity of synthetic ester and polyester degradation. J. Am. Chem. Soc. 131, 15711–15716. doi: 10.1021/ja9046697, PMID: 19810726 PMC2796240

[ref40] Liu F. Wang T. Yang W. Zhang Y. Gong Y. Fan X.. (2023). Current advances in the structural biology and molecular engineering of PETase. Front. Bioeng. Biotechnol. 11:1263996. doi: 10.3389/fbioe.2023.1263996, PMID: 37795175 PMC10546322

[ref41] Liu J. Xu G. Dong W. Xu N. Xin F. Ma J.. (2018). Biodegradation of diethyl terephthalate and polyethylene terephthalate by a novel identified degrader Delftia sp. WL-3 and its proposed metabolic pathway. Lett. Appl. Microbiol. 67, 254–261. doi: 10.1111/lam.13014, PMID: 29856468

[ref42] Liu R. Xu H. Zhao S. Dong C. Li J. Wei G.. (2024). Polyethylene terephthalate (PET)-degrading bacteria in the pelagic deep-sea sediments of the Pacific Ocean. Environ. Pollut. 352:124131. doi: 10.1016/j.envpol.2024.124131, PMID: 38734049

[ref43] Lu R. Cao L. Wang K. Ledesma-Amaro R. Ji X. J. (2021). Engineering Yarrowia lipolytica to produce advanced biofuels: current status and perspectives. Bioresour. Technol. 341:125877. doi: 10.1016/j.biortech.2021.125877, PMID: 34523574

[ref44] Maheswaran B. Al-Ansari M. Al-Humaid L. Sebastin Raj J. Kim W. Karmegam N.. (2023). In vivo degradation of polyethylene terephthalate using microbial isolates from plastic polluted environment. Chemosphere 310:136757. doi: 10.1016/j.chemosphere.2022.136757, PMID: 36228720

[ref45] Makryniotis K. Nikolaivits E. Gkountela C. Vouyiouka S. Topakas E. (2023). Discovery of a polyesterase from Deinococcus maricopensis and comparison to the benchmark LCCICCG suggests high potential for semi-crystalline post-consumer PET degradation. J. Hazard. Mater. 455:131574. doi: 10.1016/j.jhazmat.2023.131574, PMID: 37150100

[ref46] Malafatti-Picca L. Bucioli E. C. de Barros Chaves M. R. de Castro A. M. Valoni É. de Oliveira V. M.. (2023). Fungal screening for potential PET depolymerization. Polymers (Basel) 15:1581. doi: 10.3390/polym15061581, PMID: 36987362 PMC10053415

[ref47] Malafatti-Picca L. de Barros Chaves M. R. de Castro A. M. Valoni É. de Oliveira V. M. Marsaioli A. J.. (2019). Hydrocarbon-associated substrates reveal promising fungi for poly (ethylene terephthalate) (PET) depolymerization. Braz. J. Microbiol. 50, 633–648. doi: 10.1007/s42770-019-00093-3, PMID: 31175657 PMC6863199

[ref48] Maurya A. C. Bhattacharya A. Khare S. K. (2024). Biodegradation of terephthalic acid using *Rhodococcus erythropolis* MTCC 3951: insights into the degradation process, applications in wastewater treatment and polyhydroxyalkanoate production. Environ. Sci. Pollut. Res. Int. 31, 57376–57385. doi: 10.1007/s11356-023-30054-1, PMID: 37794223

[ref49] Moyses D. N. Teixeira D. A. Waldow V. A. Freire D. M. G. Castro A. M. (2021). Fungal and enzymatic bio-depolymerization of waste post-consumer poly(ethylene terephthalate) (PET) bottles using Penicillium species. 3 Biotech 11:435. doi: 10.1007/s13205-021-02988-1, PMID: 34603913 PMC8446152

[ref50] Mrigwani A. Pitaliya M. Kaur H. Kasilingam B. Thakur B. Guptasarma P. (2023). Rational mutagenesis of *Thermobifida fusca* cutinase to modulate the enzymatic degradation of polyethylene terephthalate. Biotechnol. Bioeng. 120, 674–686. doi: 10.1002/bit.28305, PMID: 36514261

[ref51] Nazari M. T. Simon V. Machado B. S. Crestani L. Marchezi G. Concolato G.. (2022). Rhodococcus: a promising genus of actinomycetes for the bioremediation of organic and inorganic contaminants. J. Environ. Manag. 323:116220. doi: 10.1016/j.jenvman.2022.116220, PMID: 36116255

[ref52] Nyyssölä A. Pihlajaniemi V. Järvinen R. Mikander S. Kontkanen H. Kruus K.. (2013). Screening of microbes for novel acidic cutinases and cloning and expression of an acidic cutinase from *Aspergillus niger* CBS 513.88. Enzym. Microb. Technol. 52, 272–278. doi: 10.1016/j.enzmictec.2013.01.005, PMID: 23540930

[ref53] Ostle C. Thompson R. C. Broughton D. Gregory L. Wootton M. Johns D. G. (2019). The rise in ocean plastics evidenced from a 60-year time series. Nat. Commun. 10:1622. doi: 10.1038/s41467-019-09506-1, PMID: 30992426 PMC6467903

[ref54] Park Y. K. Ledesma-Amaro R. (2023). What makes Yarrowia lipolytica well suited for industry? Trends Biotechnol. 41, 242–254. doi: 10.1016/j.tibtech.2022.07.006, PMID: 35940976

[ref55] Perez-Garcia P. Chow J. Costanzi E. Gurschke M. Dittrich J. Dierkes R. F.. (2023). An archaeal lid-containing feruloyl esterase degrades polyethylene terephthalate. Commun. Chem. 6:193. doi: 10.1038/s42004-023-00998-z, PMID: 37697032 PMC10495362

[ref56] Pires C. S. Costa L. Barbosa S. G. Sequeira J. C. Cachetas D. Freitas J. P.. (2024). Microplastics biodegradation by estuarine and landfill microbiomes. Microb. Ecol. 87:88. doi: 10.1007/s00248-024-02399-8, PMID: 38943017 PMC11213754

[ref57] Qi X. Yan W. Cao Z. Ding M. Yuan Y. (2021). Current advances in the biodegradation and bioconversion of polyethylene terephthalate. Microorganisms 10:39. doi: 10.3390/microorganisms10010039, PMID: 35056486 PMC8779501

[ref58] Qiu J. Chen Y. Zhang L. Wu J. Zeng X. Shi X.. (2024). A comprehensive review on enzymatic biodegradation of polyethylene terephthalate. Environ. Res. 240:117427. doi: 10.1016/j.envres.2023.117427, PMID: 37865324

[ref59] Radley E. Davidson J. Foster J. Obexer R. Bell E. L. Green A. P. (2023). Engineering enzymes for environmental sustainability. Angew. Chem. Int. Ed. Engl. 62:e202309305. doi: 10.1002/anie.202309305, PMID: 37651344 PMC10952156

[ref60] Ragaert K. Delva L. Van Geem K. (2017). Mechanical and chemical recycling of solid plastic waste. Waste Manag. 69, 24–58. doi: 10.1016/j.wasman.2017.07.044, PMID: 28823699

[ref61] Roman E. K. B. Ramos M. A. Tomazetto G. Foltran B. B. Galvão M. H. Ciancaglini I.. (2024). Plastic-degrading microbial communities reveal novel microorganisms, pathways, and biocatalysts for polymer degradation and bioplastic production. Sci. Total Environ. 949:174876. doi: 10.1016/j.scitotenv.2024.174876, PMID: 39067601

[ref62] Sahu S. Kaur A. Khatri M. Singh G. Arya S. K. (2023). A review on cutinases enzyme in degradation of microplastics. J. Environ. Manag. 347:119193. doi: 10.1016/j.jenvman.2023.119193, PMID: 37797518

[ref63] Sales J. C. S. de Castro A. M. Ribeiro B. D. Coelho M. A. Z. (2021). Improved production of biocatalysts by Yarrowia lipolytica using natural sources of the biopolyesters cutin and suberin, and their application in hydrolysis of poly (ethylene terephthalate) (PET). Bioprocess Biosyst. Eng. 44, 2277–2287. doi: 10.1007/s00449-021-02603-w, PMID: 34165618

[ref64] Sanniyasi E. Gopal R. K. Gunasekar D. K. Raj P. P. (2021). Biodegradation of low-density polyethylene (LDPE) sheet by microalga, Uronema africanum Borge. Sci. Rep. 11:17233. doi: 10.1038/s41598-021-96315-6, PMID: 34446729 PMC8390665

[ref65] Sarmah P. Rout J. (2018). Efficient biodegradation of low-density polyethylene by cyanobacteria isolated from submerged polyethylene surface in domestic sewage water. Environ. Sci. Pollut. Res. Int. 25, 33508–33520. doi: 10.1007/s11356-018-3079-7, PMID: 30267347

[ref66] Satta A. Zampieri G. Loprete G. Campanaro S. Treu L. Bergantino E. (2024). Metabolic and enzymatic engineering strategies for polyethylene terephthalate degradation and valorization. Rev. Environ. Sci. Biotechnol. 23, 351–383. doi: 10.1007/s11157-024-09688-1

[ref67] Shirke A. N. Basore D. Butterfoss G. L. Bonneau R. Bystroff C. Gross R. A. (2016). Toward rational thermostabilization of aspergillus oryzae cutinase: insights into catalytic and structural stability. Proteins 84, 60–72. doi: 10.1002/prot.24955, PMID: 26522152 PMC4715774

[ref68] Singh Jadaun J. Bansal S. Sonthalia A. Rai A. K. Singh S. P. (2022). Biodegradation of plastics for sustainable environment. Bioresour. Technol. 347:126697. doi: 10.1016/j.biortech.2022.126697, PMID: 35026422

[ref69] Sui B. Wang T. Fang J. Hou Z. Shu T. Lu Z.. (2023). Recent advances in the biodegradation of polyethylene terephthalate with cutinase-like enzymes. Front. Microbiol. 14:1265139. doi: 10.3389/fmicb.2023.1265139, PMID: 37849919 PMC10577388

[ref70] Sun T. Yu Y. Wang K. Ledesma-Amaro R. Ji X. J. (2021). Engineering Yarrowia lipolytica to produce fuels and chemicals from xylose: a review. Bioresour. Technol. 337:125484. doi: 10.1016/j.biortech.2021.125484, PMID: 34320765

[ref71] Świderek K. Velasco-Lozano S. Galmés M. À. Olazabal I. Sardon H. López-Gallego F.. (2023). Mechanistic studies of a lipase unveil effect of pH on hydrolysis products of small PET modules. Nat. Commun. 14:3556. doi: 10.1038/s41467-023-39201-1, PMID: 37321996 PMC10272158

[ref72] Temporiti M. E. E. Nicola L. Nielsen E. Tosi S. (2022). Fungal enzymes involved in plastics biodegradation. Microorganisms 10:1180. doi: 10.3390/microorganisms10061180, PMID: 35744698 PMC9230134

[ref73] Tournier V. Topham C. M. Gilles A. David B. Folgoas C. Moya-Leclair E.. (2020). An engineered PET depolymerase to break down and recycle plastic bottles. Nature 580, 216–219. doi: 10.1038/s41586-020-2149-4, PMID: 32269349

[ref74] Wang H. Zhu J. Sun M. Gu M. Xie X. Ying T.. (2025). Biodegradation of combined pollutants of polyethylene terephthalate and phthalate esters by esterase-integrated Pseudomonas sp. JY-Q with surface-co-displayed PETase and MHETase. Synth. Syst. Biotechnol. 10, 10–22. doi: 10.1016/j.synbio.2024.08.001, PMID: 39206086 PMC11350496

[ref75] Yoshida S. Hiraga K. Takehana T. Taniguchi I. Yamaji H. Maeda Y.. (2016). A bacterium that degrades and assimilates poly(ethylene terephthalate). Science 351, 1196–1199. doi: 10.1126/science.aad6359, PMID: 26965627

[ref76] Zampolli J. Orro A. Vezzini D. Di Gennaro P. (2022). Genome-based exploration of Rhodococcus species for plastic-degrading genetic determinants using bioinformatic analysis. Microorganisms 10:1846. doi: 10.3390/microorganisms10091846, PMID: 36144448 PMC9506104

[ref77] Zampolli J. Zeaiter Z. Di Canito A. Di Gennaro P. (2019). Genome analysis and -omics approaches provide new insights into the biodegradation potential of Rhodococcus. Appl. Microbiol. Biotechnol. 103, 1069–1080. doi: 10.1007/s00253-018-9539-7, PMID: 30554387

[ref78] Zeng C. Ding F. Zhou J. Dong W. Cui Z. Yan X. (2023). Biodegradation of poly(ethylene terephthalate) by *Bacillus safensis* YX8. Int. J. Mol. Sci. 24:16434. doi: 10.3390/ijms242216434, PMID: 38003625 PMC10671283

[ref79] Zhang H. Perez-Garcia P. Dierkes R. F. Applegate V. Schumacher J. Chibani C. M.. (2021). The bacteroidetes *Aequorivita* sp. and *Kaistella jeonii* produce promiscuous esterases with PET-hydrolyzing activity. Front. Microbiol. 12:803896. doi: 10.3389/fmicb.2021.803896, PMID: 35069509 PMC8767016

[ref80] Zhao S. Liu R. Wang J. Lv S. Zhang B. Dong C.. (2023). Biodegradation of polyethylene terephthalate (PET) by diverse marine bacteria in deep-sea sediments. Environ. Microbiol. 25, 2719–2731. doi: 10.1111/1462-2920.16460, PMID: 37421171

